# Supplementary material for: Simulated Docking Predicts Putative Channels for the Transport of Long-Chain Fatty Acids in Vibrio cholerae
Source: Biomolecules. 2022 Sep 9;12(9):1269. doi: 10.3390/biom12091269 (PMC9496633; doi:10.3390/biom12091269)
Supplement: Supplementary file 1 [file biomolecules-12-01269-s001.zip › biomolecules-1780725-supplementary.pdf]

biomolecules

MDPI

Article

Simulated Docking Predicts Putative Channels for the Transport of Long-Chain Fatty Acids in *Vibrio cholerae*

Andrew Turgeson [1,†], Lucas Morley [2,†], David Giles [3,†] and Bradley Harris [1,*,†]

1 Department of Chemical Engineering, University of Tennessee at Chattanooga, Chattanooga, TN 37403, USA
2 Department of Biological Sciences, University of Pittsburgh, Pittsburgh, PA 15260, USA
3 Department of Biology, Geology, and Environmental Science, University of Tennessee at Chattanooga, Chattanooga, TN 37403, USA
* Correspondence: bradley-harris@utc.edu; Tel.: +1-423-425-2209
† These authors contributed equally to this work.

check for updates

Citation: Turgeson, A.; Morley, L.; Giles, D.; Harris, B. Simulated Docking Predicts Putative Channels for the Transport of Long-Chain Fatty Acids in *Vibrio cholerae*. *Biomolecules* **2022**, *12*, 1269. https://doi.org/10.3390/biom12091269

Academic Editor: Laurent Soulère

Received: 4 June 2022
Accepted: 1 September 2022
Published: 9 September 2022

Publisher's Note: MDPI stays neutral with regard to jurisdictional claims in published maps and institutional affiliations.

Copyright: © 2022 by the authors. Licensee MDPI, Basel, Switzerland. This article is an open access article distributed under the terms and conditions of the Creative Commons Attribution (CC BY) license (https://creativecommons.org/licenses/by/4.0/).

1. Supplemental Material

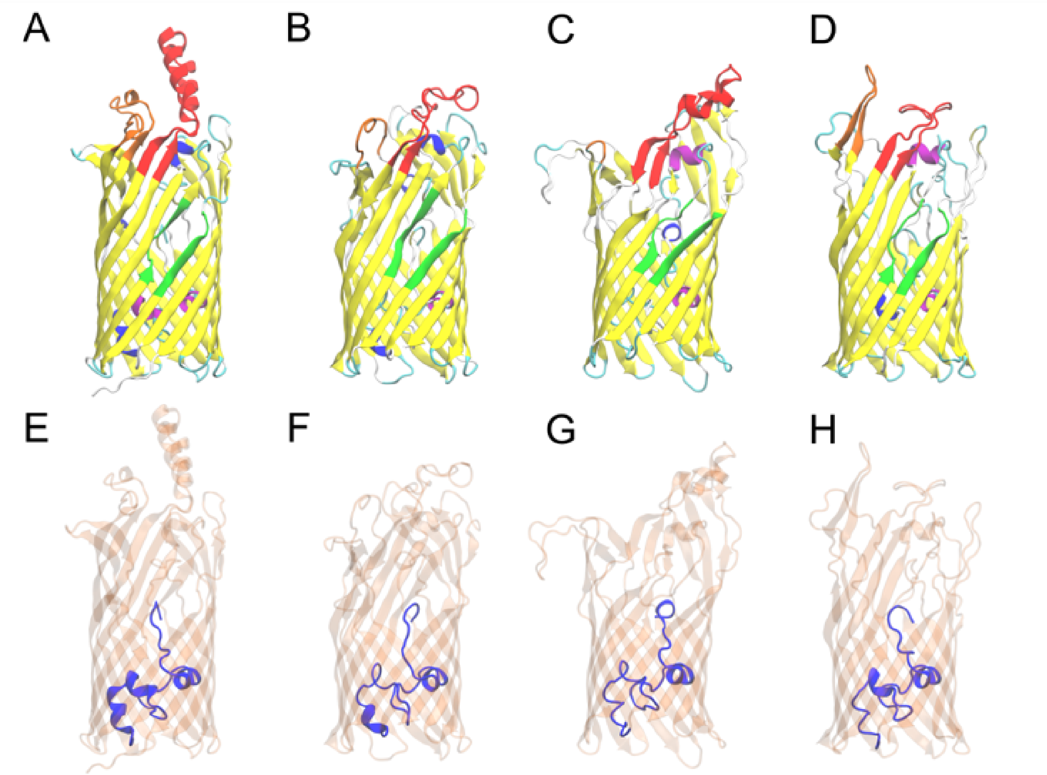

Figure S1. I-TASSER folded structures. (A) the *E. coli b2344* 1T16 crystal strcuture compared to (B) *vc1042* (C), *vc1043*, and (D) *vca0862*. L3 and L4 loops are colored red and orange respectively, and the S3 kink is green for reference. (E-H) are views of the N-terminal hatch domain (blue) for *E. coli b2344*, *vc1042*, *vc1043*, and *vca0862* respectively

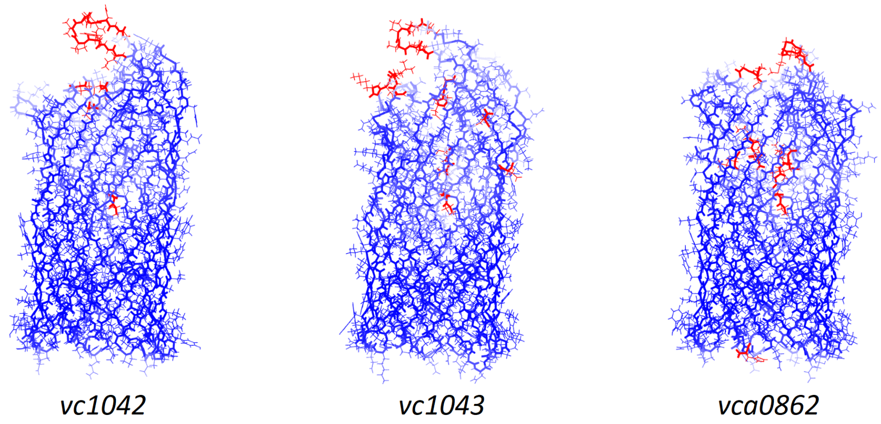

Figure S2. AlphaFold generated structures. The structures are colored by pLDDT scaling from 100 (blue) to 90 (white). Any residues that were below 90 were colored red. The only residues that had a pLDDT value of less than 80 were in the extracellular loops with the exception of ALA1 in homolog *vc1043* with a pLDDT of 78.75.

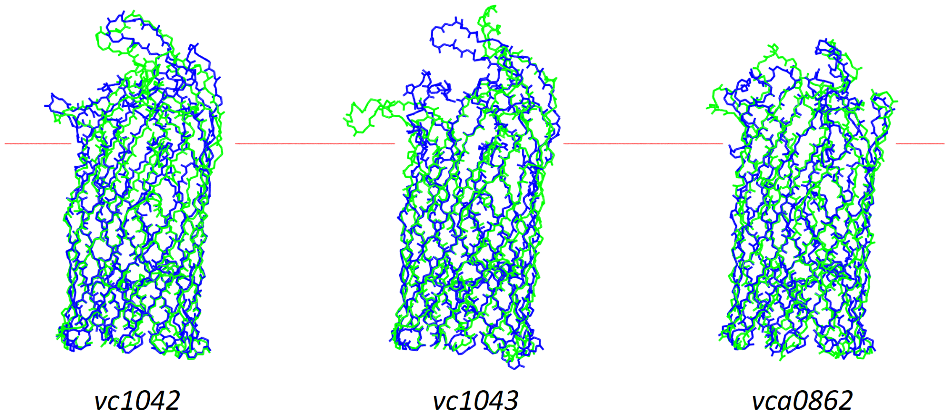

Figure S3. Aligned backbone structures generated by I-TASSER (green) and AlphaFold (blue). The red line indicates the approximate location of the start of the extracellular loops.

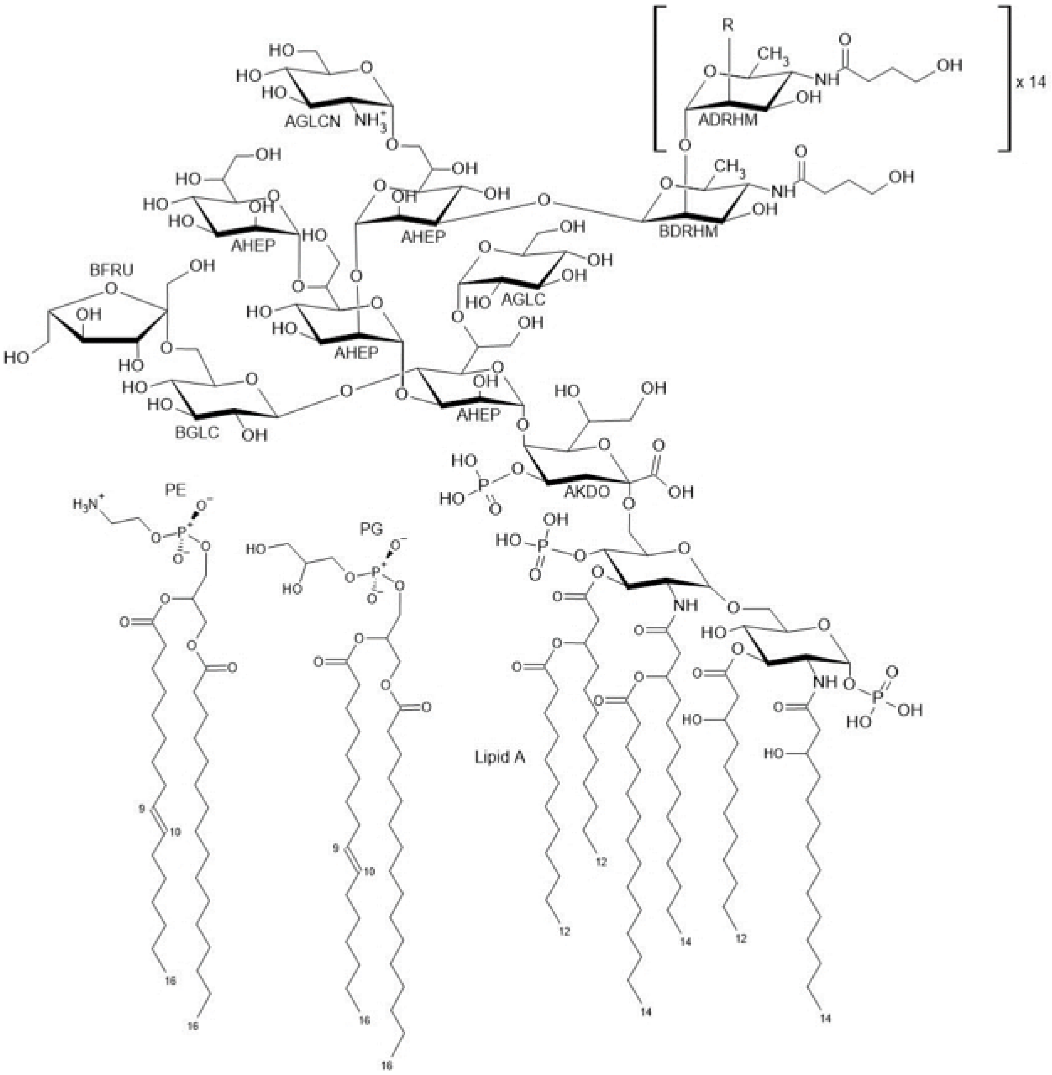

Figure S4. 2-D representations of constituents of the *V. cholerae* membranes

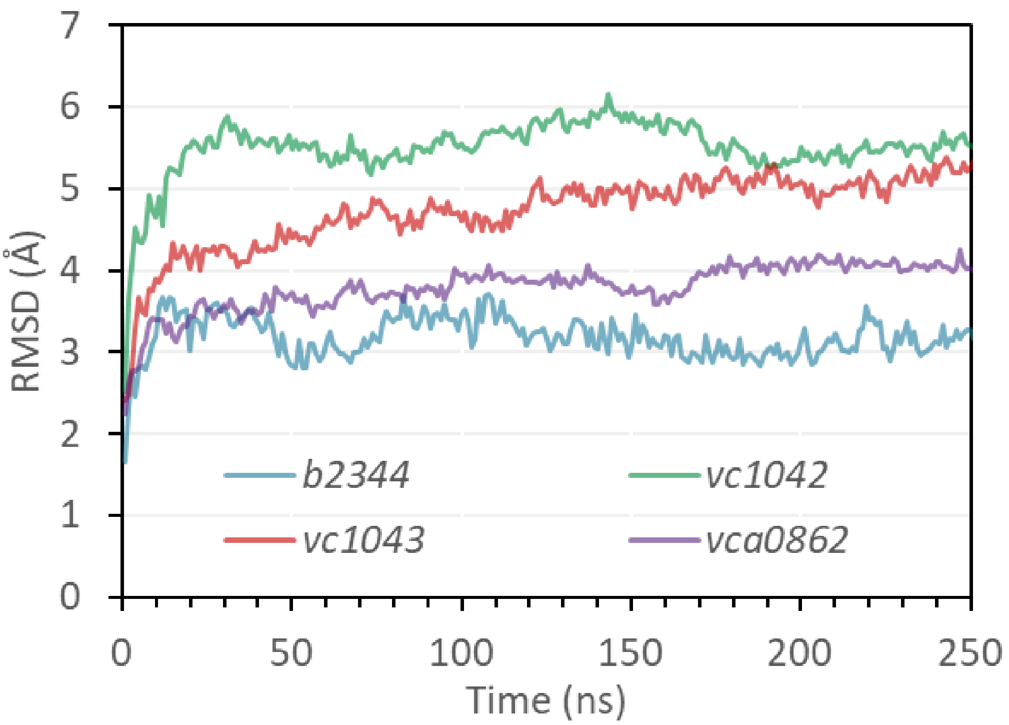

Figure S5. RMSD of equilibrated systems over the 250 ns trajectory.

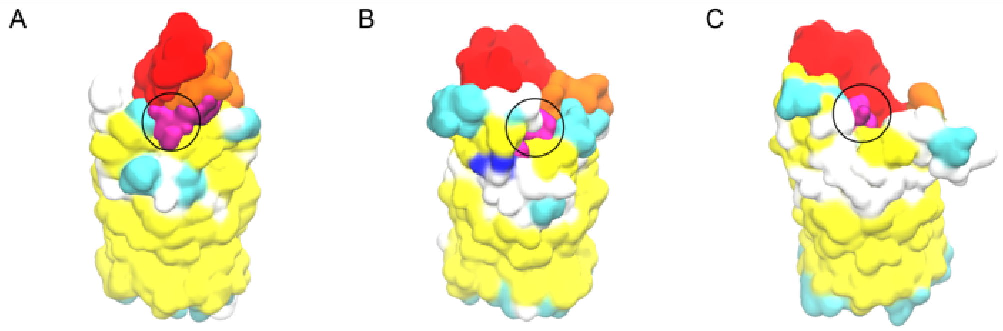

Figure S6. Surface view of FA transport channel entrance points (shown in purple) for (A) *b2344*, (B) *vc1042*, (C) *vc1043* FadL proteins after equilibration. The general protein surface is colored by secondary structure and the L3 and L4 loops are colored red and orange respectively. This perspective is opposite of the S3 kink.

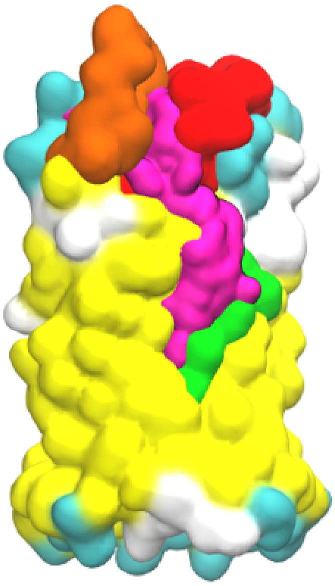

Figure S7. Surface view of FA exterior pathway (shown in purple) for *vca0862* FadL protein after equilibration. The general protein surface is colored by secondary structure, the S3 kink is colored green, and the L3 and L4 loops are colored red and orange respectively.

Table S1. Fatty acids tested during docking

Fatty Acid	Abbreviation	Biological Significance
Lauryldimethylamine oxide	LDAO	Antimicrobial zwitterionic surfactant ($C_{14}H_{31}NO$)
Palmitoleic acid	16:1	A common component of bacterial lipids
Linoleic acid	18:2	Found in VC membrane phospholipids and bile
α(alpha)-Linolenic acid	18:3α	Found in microalgae, cyanobacteria, and fish
γ(gamma)-Linolenic acid	18:3γ	An intermediate in the conversion of linoleic to arachidonic acid
Dihomo-γ(gamma)-linolenic acid	20:3	Found in microalgae and fish
Arachidonic acid	20:4	Key cellular signaling molecule and inflammatory intermediate
Eicosapentaenoic acid	20:5	Found in microalgae and fish
Tetraethylene glycol monooctyl ether	C8E4	A membrane solubilizing detergent ether ($C_{16}H_{34}O_5$)
Docosahexaenoic acid	22:6	Found in microalgae and fish

Table S2. Ramachandran Z-Scores

Homolog	I-TASSER	AlphaFold	I-TASSER Equilibrated
b2344	−2.40 ± 0.37*		−1.87 ± 0.38
vc1042	−1.23 ± 0.42	0.82 ± 0.42	−2.40 ± 0.37
vc1043	−2.04 ± 0.39	1.34 ± 0.44	−1.97 ± 0.40
vca0862	−3.85 ± 0.37	0.46 ± 0.41	−1.80 ± 0.40

* homolog *b2344* was not computationally folded, but presented for reference.

Table S3. RMSD of Initial I-TASSER and AlphaFold Backbone Structures.

**Homolog**	**RMSD**	**RMSD Excluding EC Loops**
vc1042	3.0809	1.3701
vc1043	3.8402	1.2824
vca0862	1.8902	1.2421

Table S4. Best Conformation Energies (most negative) from Docking (energy units in kcal/mol).

Homolog	LDAO	16:1	18:2	18:3α	18:3γ	20:3	20:4	20:5	C8E4	22:6
b2344 1T16	−10.11	−12.41	−13.53	−13.72	−13.33	−14.33	−14.58	−14.5	−10.96	−14.25
b2344	−9.15	−9.91	−10.36	−10.13	−10.45	−10.61	−10.71	−9.88	−9.92	−11.72
vc1042	−8.81	−10.37	−10.12	−9.9	−10.28	−11	−11.44	−10.55	−9.55	−11.24
vc1043	−9.92	−10.48	−11.48	−11.12	−11.17	−12.19	−12.37	−11.93	−10.22	−12.4
vca0862	−9.28	−10.95	−10.62	−9.93	−10.36	−10.84	−10.32	−9.96	−9.57	−10.95

Table S5. Overall Average Conformation Energy from Docking (energy units in kcal/mol).

Homolog	LDAO	16:1	18:2	18:3α	18:3γ	20:3	20:4	20:5	C8E4	22:6
b2344 1T16	−9.17	−9.62	−9.55	−9.32	−9.74	−9.83	−9.46	−9.44	−8.15	−9.95
b2344	−7.39	−7.82	−8.09	−7.87	−8.18	−8.42	−8.34	−8.2	−7.47	−8.69
vc1042	−7.86	−8.33	−8.56	−8.36	−8.62	−9.01	−8.77	−8.7	−7.77	−9.24
vc1043	−8.53	−9.07	−9.42	−9.1	−9.38	−9.82	−9.61	−9.42	−8.17	−10.11
vca0862	−7.57	−7.81	−7.97	−7.6	−7.94	−8.22	−7.94	−7.83	−7.14	−8.21
