# Supplementary material for: Simulated Docking Predicts Putative Channels for the Transport of Long-Chain Fatty Acids in Vibrio cholerae
Source: Biomolecules. 2022 Sep 9;12(9):1269. doi: 10.3390/biom12091269 (PMC9496633; doi:10.3390/biom12091269)
Supplement: Supplementary file 1 [file biomolecules-12-01269-s001.zip › Definitions/logo-mdpi-eps-converted-to.pdf]

MDPI
