# Supplementary figures and images for: Simulated Docking Predicts Putative Channels for the Transport of Long-Chain Fatty Acids in Vibrio cholerae
Source: Biomolecules. 2022 Sep 9;12(9):1269. doi: 10.3390/biom12091269 (PMC9496633; doi:10.3390/biom12091269)

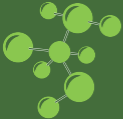

*biomolecules*

Supplement: Supplementary file 1 [file biomolecules-12-01269-s001.zip › Definitions/biomolecules-logo-eps-converted-to.pdf]

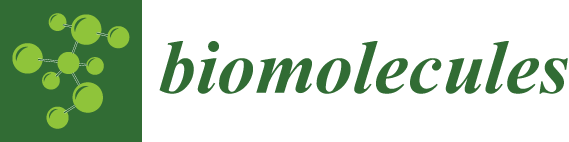

Supplement: Supplementary file 1 [file biomolecules-12-01269-s001.zip › Definitions/biomolecules-logo.png]

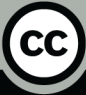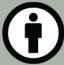

BY

Supplement: Supplementary file 1 [file biomolecules-12-01269-s001.zip › Definitions/logo-ccby-eps-converted-to.pdf]

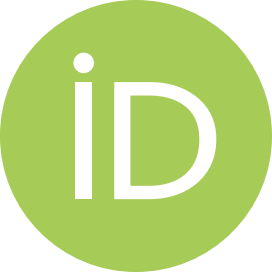

Supplement: Supplementary file 1 [file biomolecules-12-01269-s001.zip › Definitions/logo-orcid.pdf]

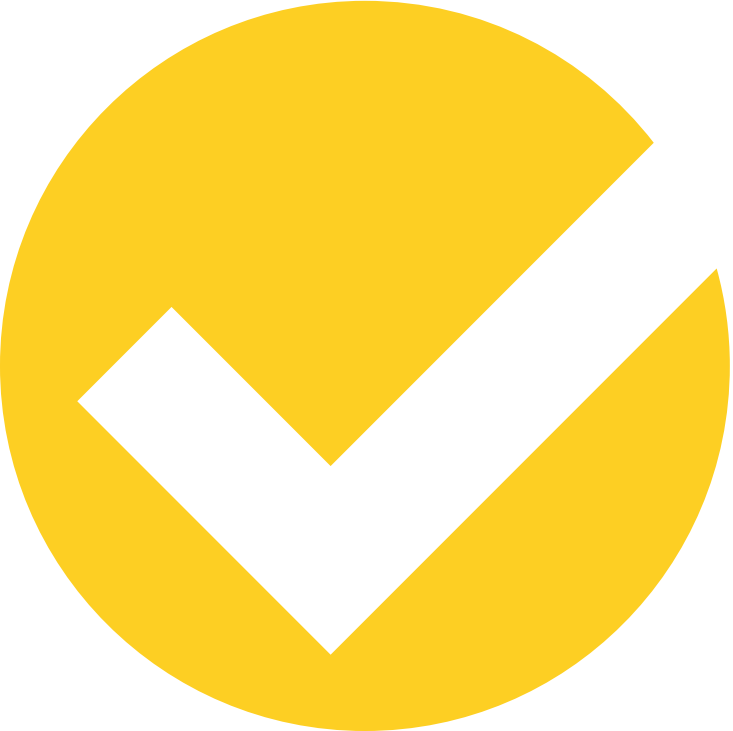

check for  
updates

Supplement: Supplementary file 1 [file biomolecules-12-01269-s001.zip › Definitions/logo-updates-eps-converted-to.pdf]

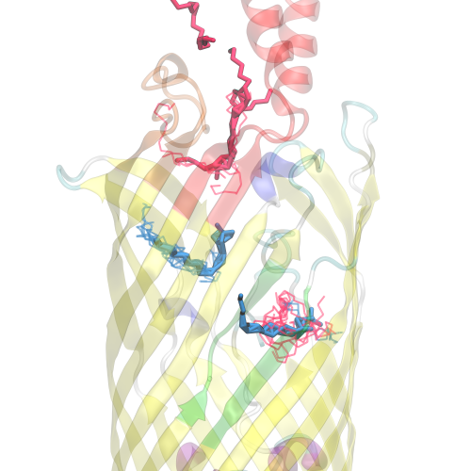

Supplement: Supplementary file 1 [file biomolecules-12-01269-s001.zip › images/1t16_dock_compare.png]

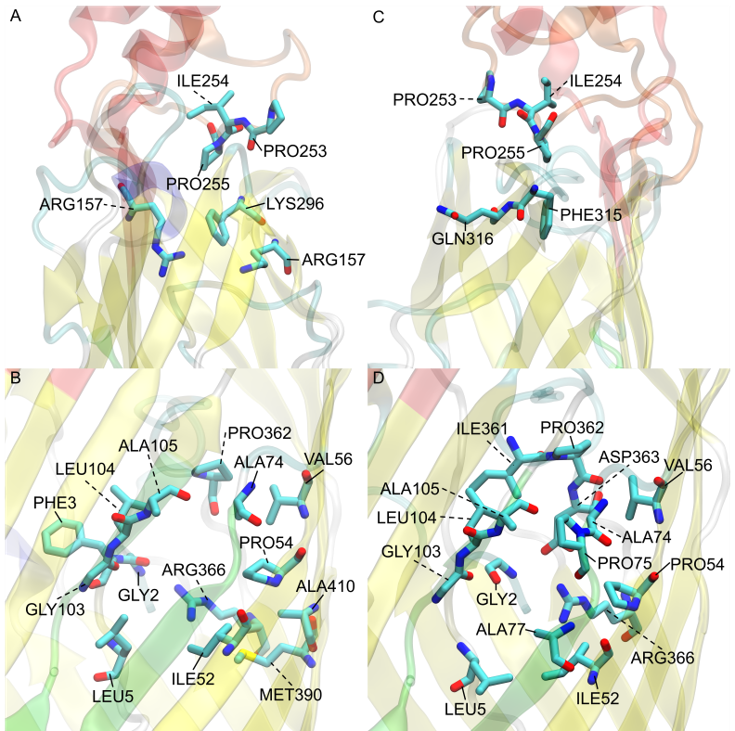

Supplement: Supplementary file 1 [file biomolecules-12-01269-s001.zip › images/1t16_ecoli_residues.png]

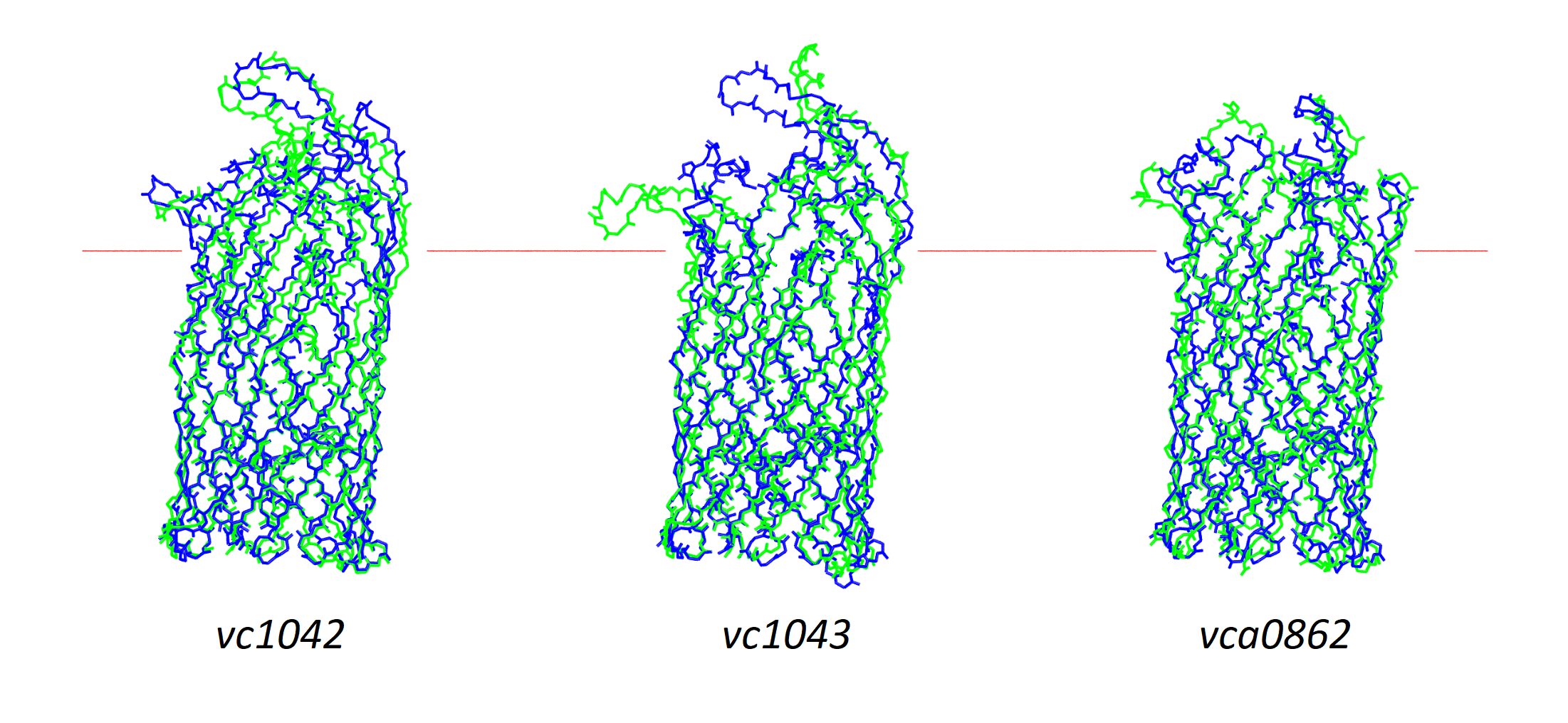

Supplement: Supplementary file 1 [file biomolecules-12-01269-s001.zip › images/AlphaFoldRMSD.png]

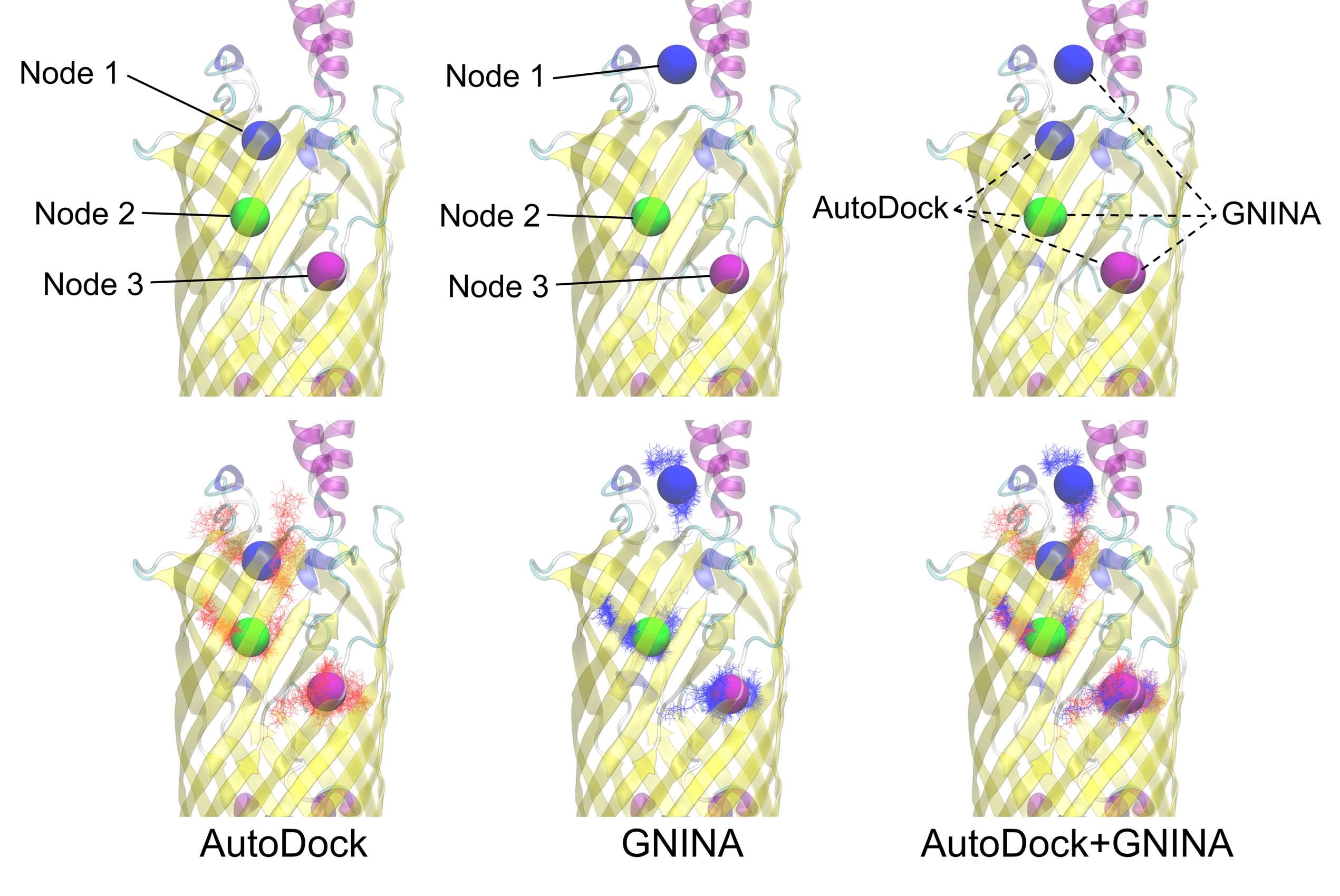

Supplement: Supplementary file 1 [file biomolecules-12-01269-s001.zip › images/AutoDockGninaCompare.png]

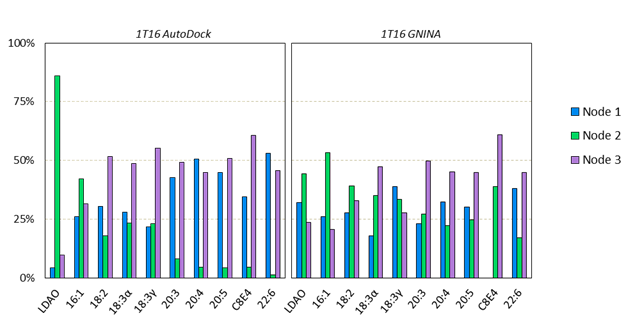

Supplement: Supplementary file 1 [file biomolecules-12-01269-s001.zip › images/AutoDock_GNINA_FA_Nodes_1t16.png]

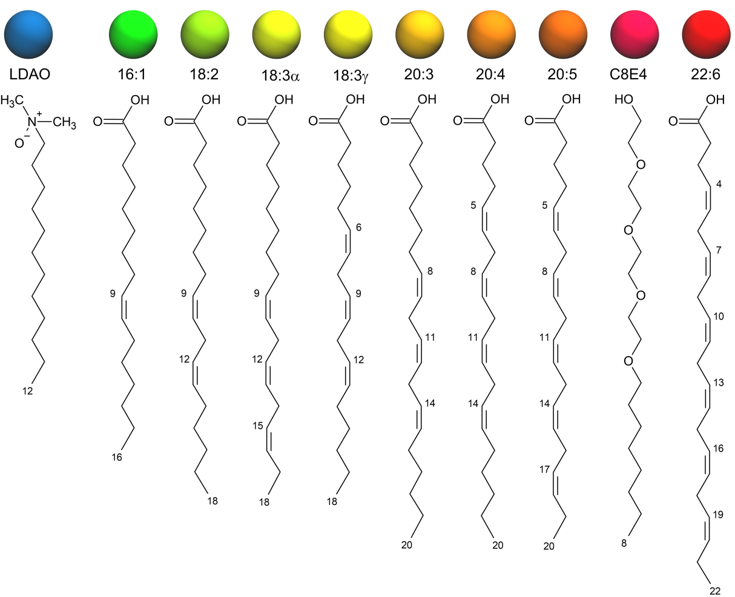

Supplement: Supplementary file 1 [file biomolecules-12-01269-s001.zip › images/FA_colors.png]

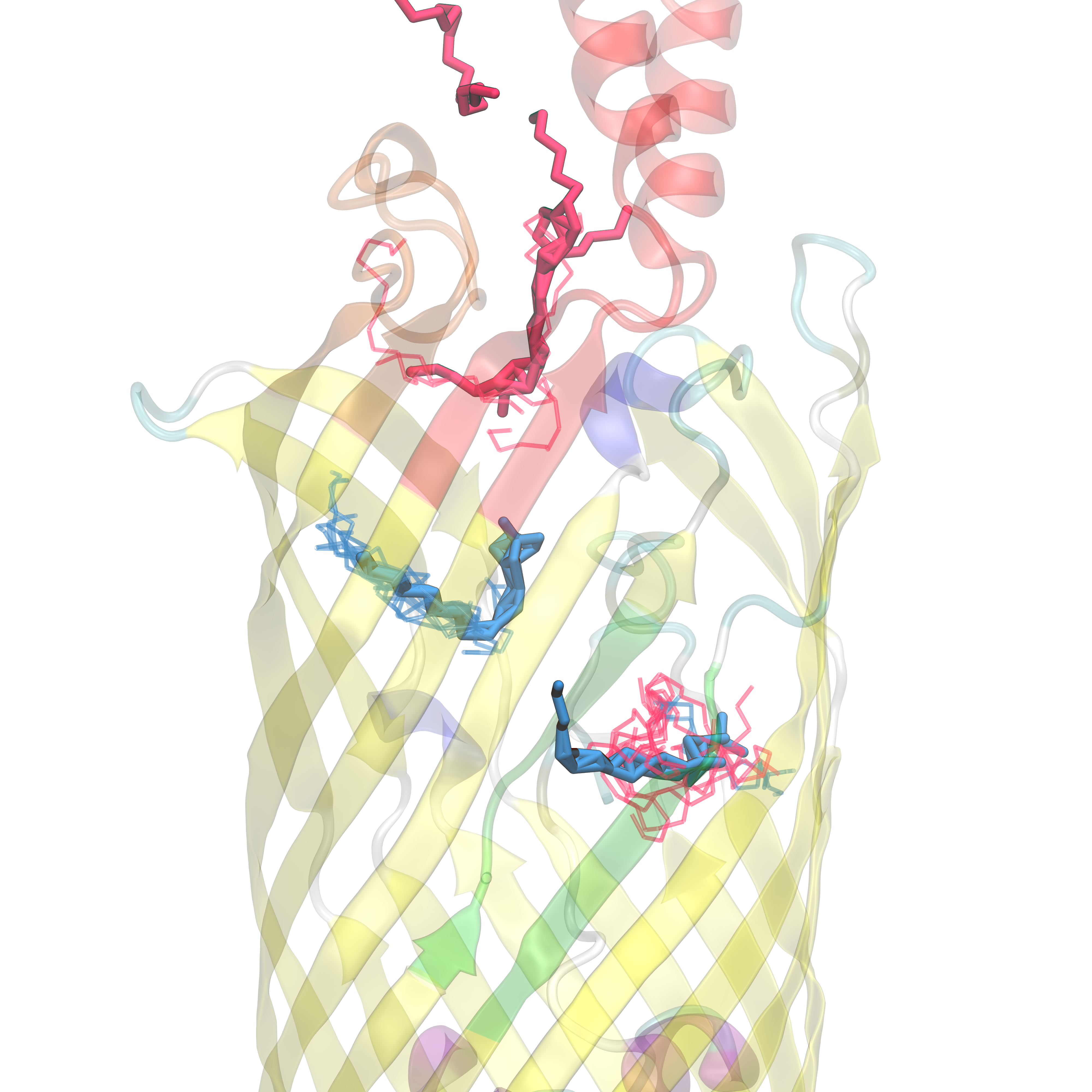

Supplement: Supplementary file 1 [file biomolecules-12-01269-s001.zip › images/HD_1t16_dock_compare.png]

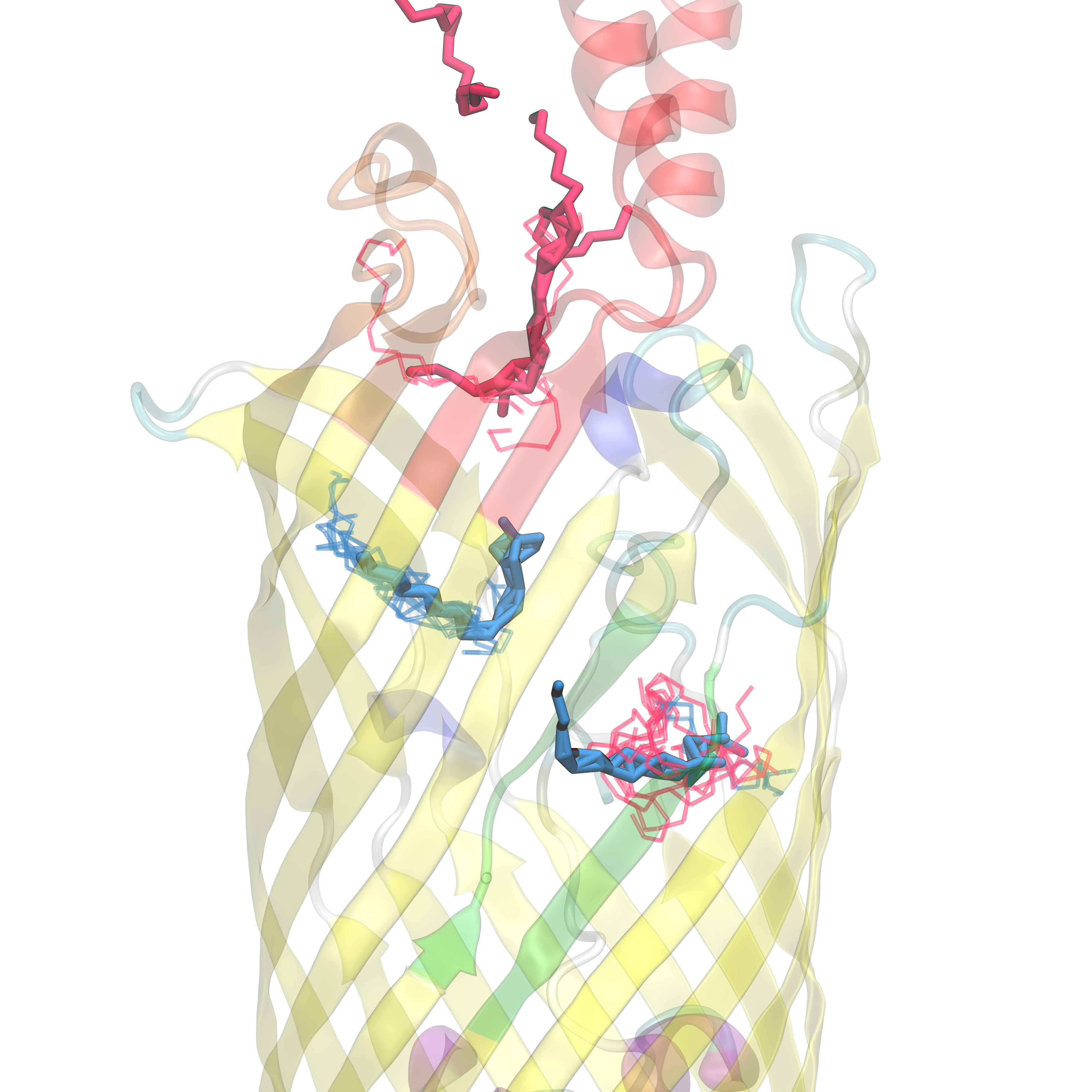

Supplement: Supplementary file 1 [file biomolecules-12-01269-s001.zip › images/HD_1t16_dock_compare.tif]

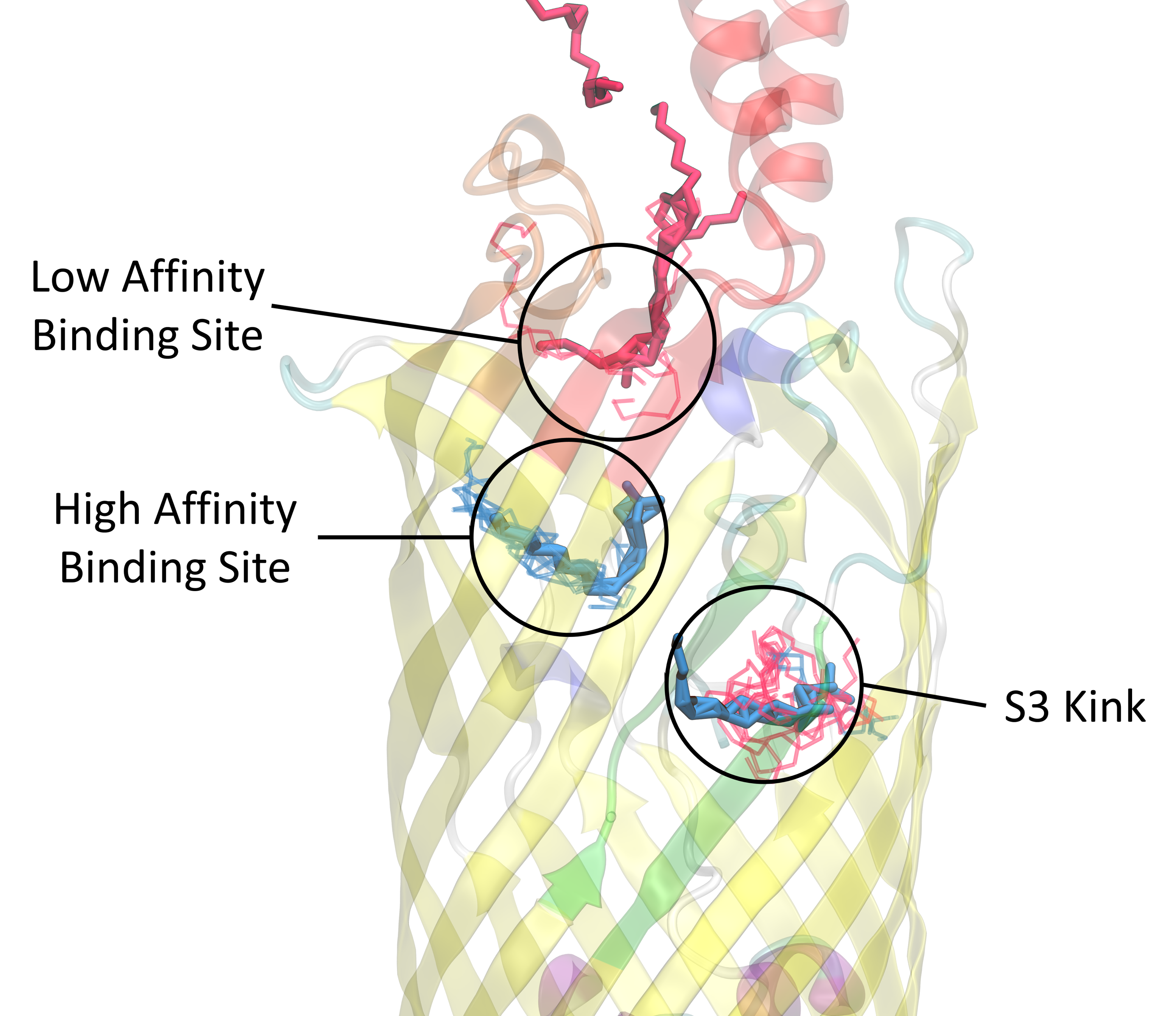

Supplement: Supplementary file 1 [file biomolecules-12-01269-s001.zip › images/HD_compare_labels.png]

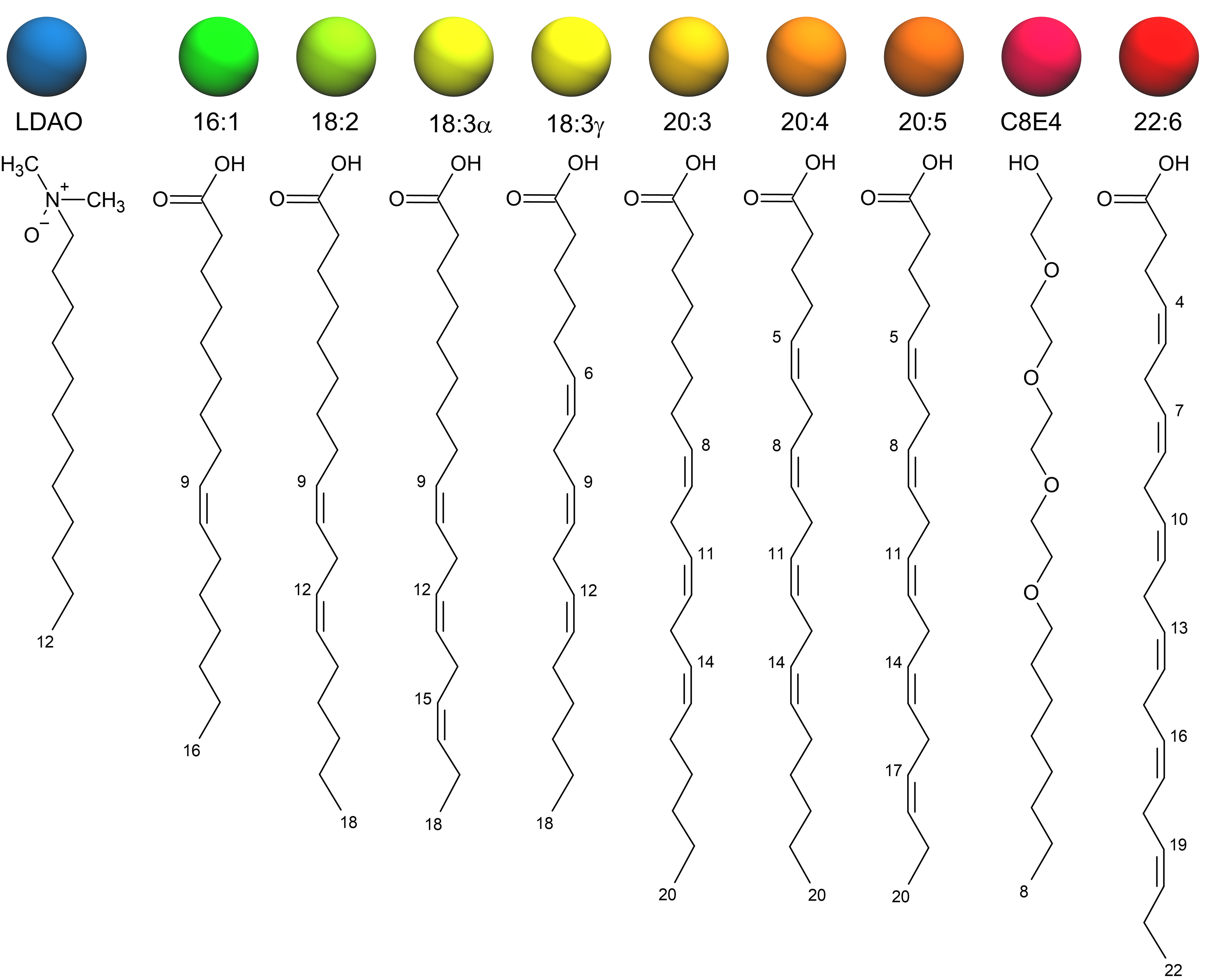

Supplement: Supplementary file 1 [file biomolecules-12-01269-s001.zip › images/HD_FA_colors.png]

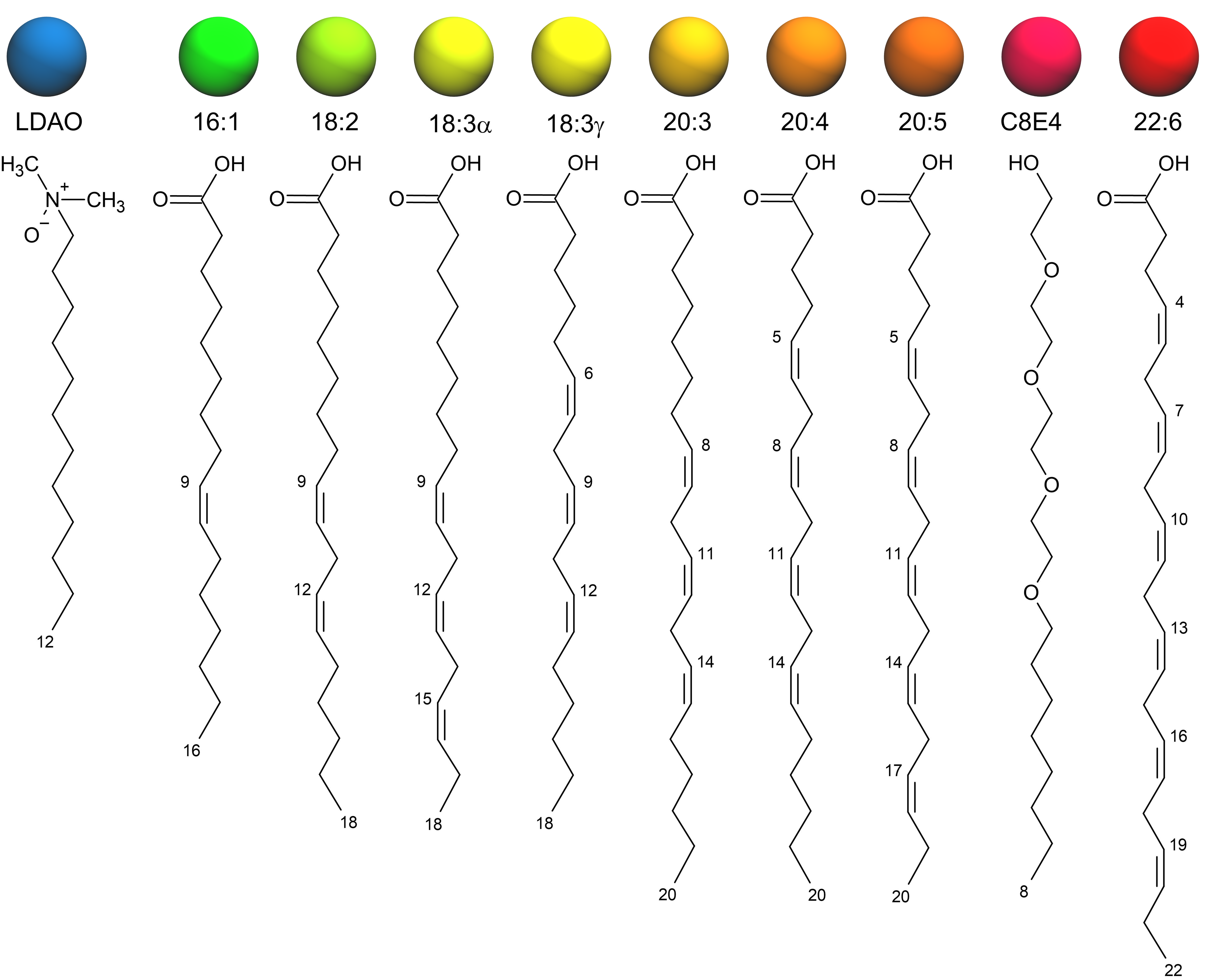

Supplement: Supplementary file 1 [file biomolecules-12-01269-s001.zip › images/HD_FA_colors.tif]

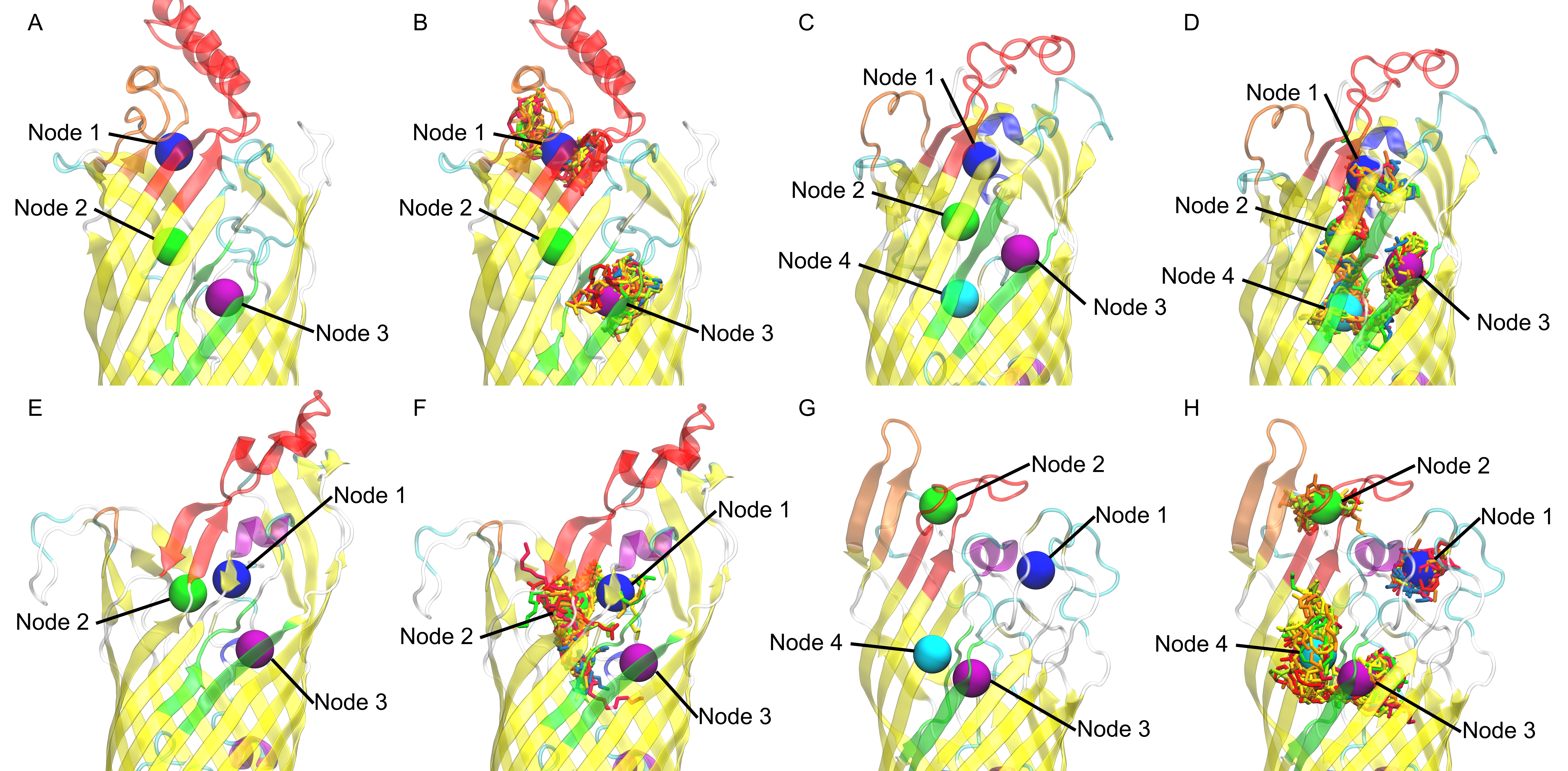

Supplement: Supplementary file 1 [file biomolecules-12-01269-s001.zip › images/HD_nodes_all.tif]

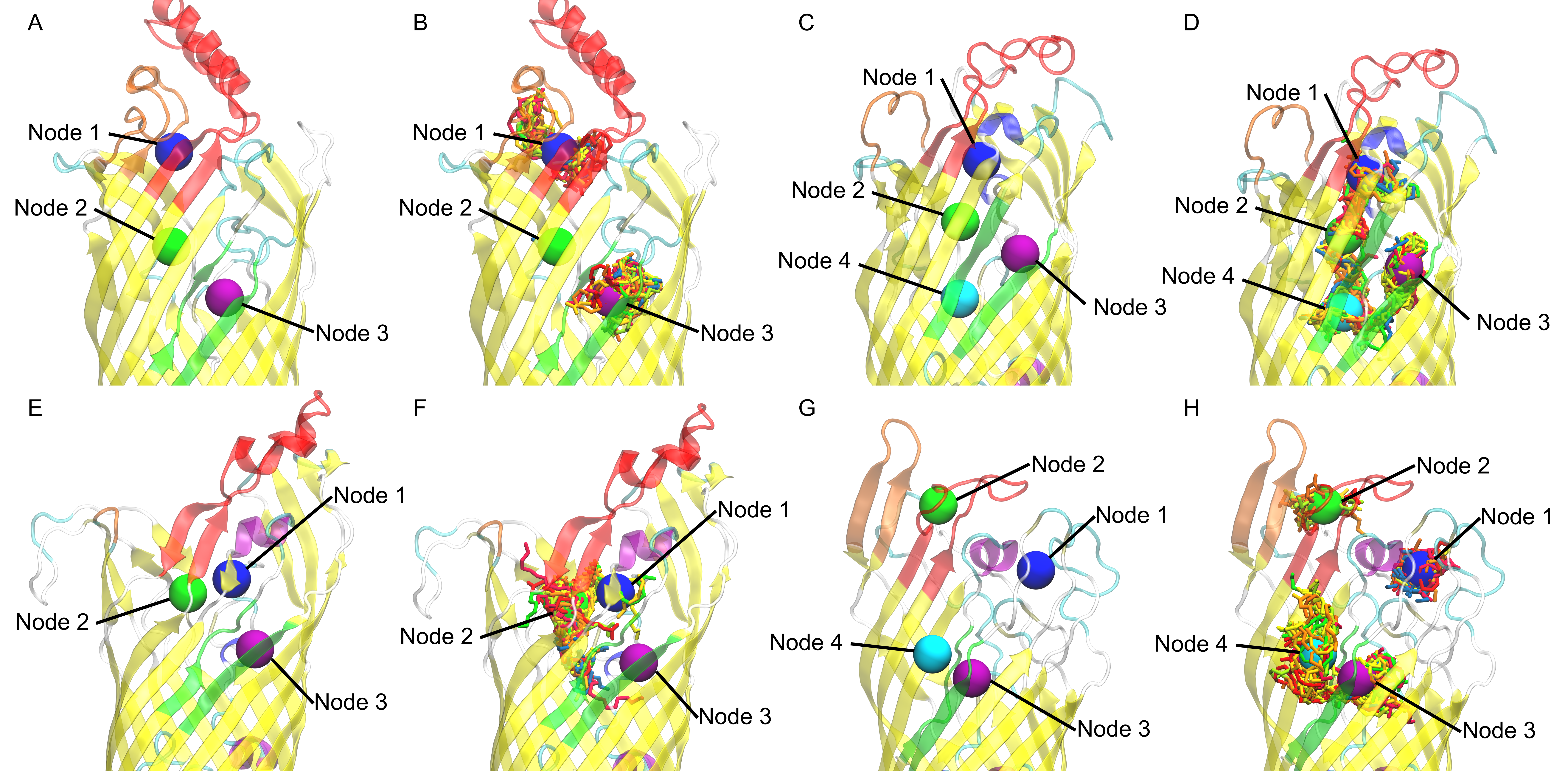

Supplement: Supplementary file 1 [file biomolecules-12-01269-s001.zip › images/HD_nodes_vc.png]

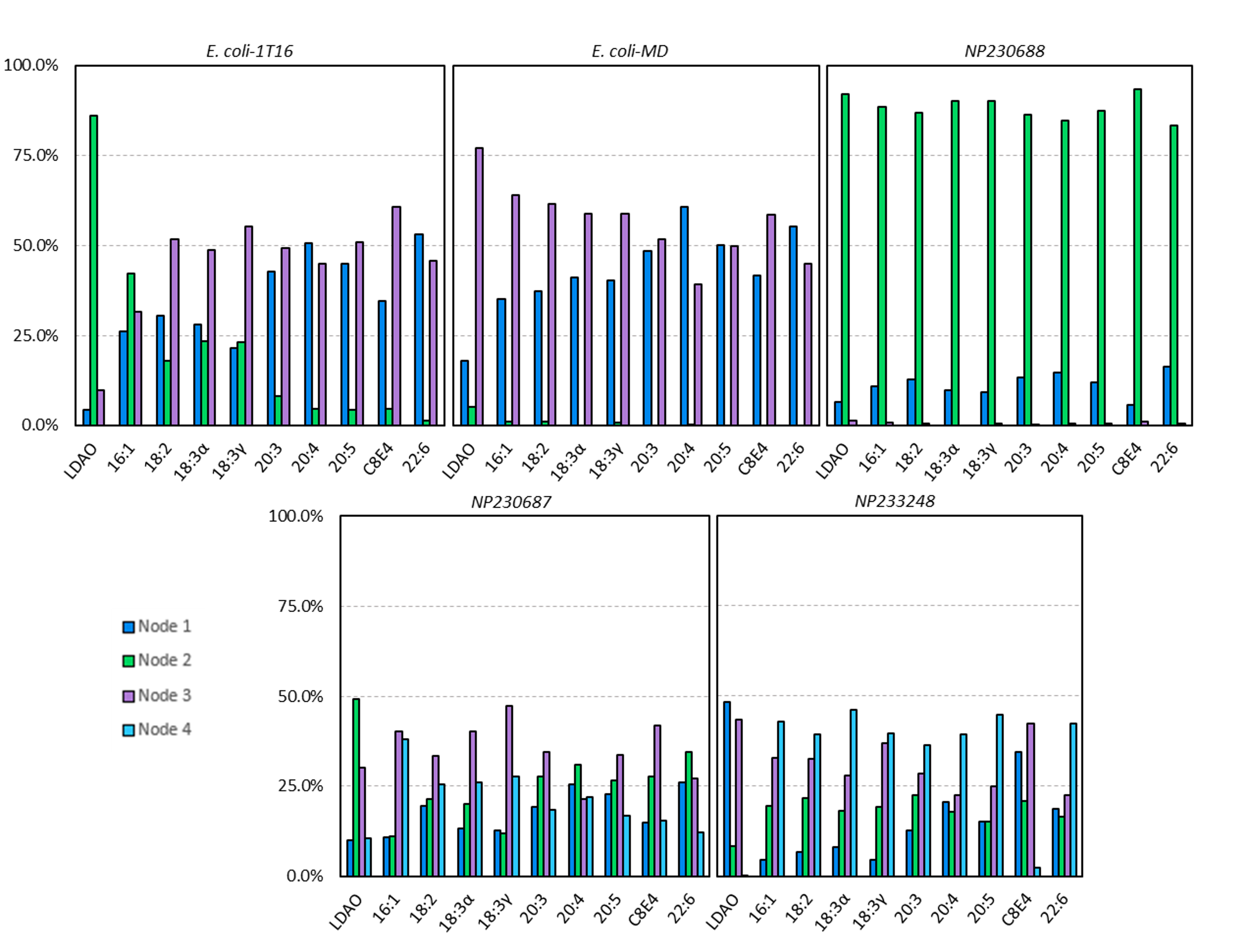

Supplement: Supplementary file 1 [file biomolecules-12-01269-s001.zip › images/HD_Node_Chart.tif]

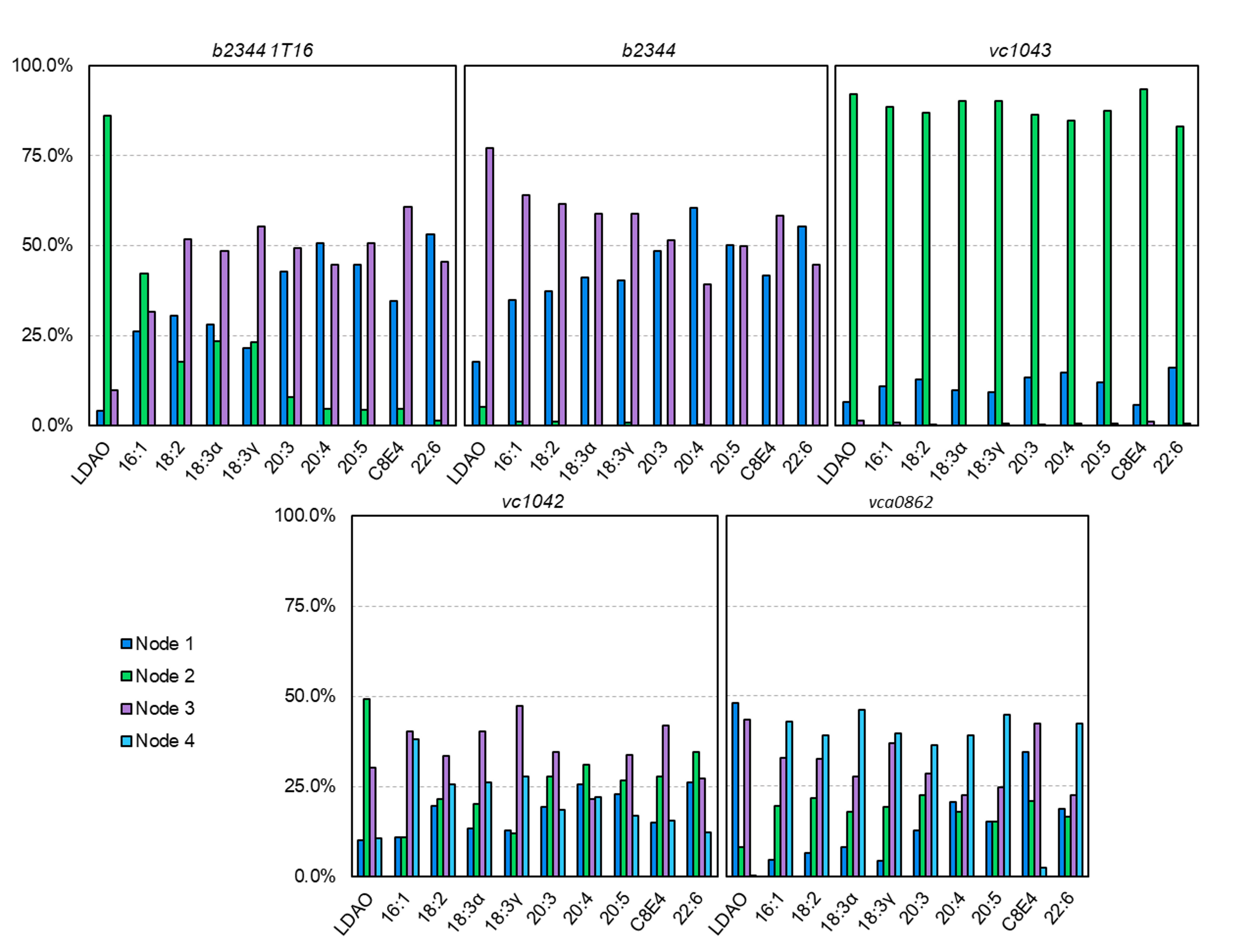

Supplement: Supplementary file 1 [file biomolecules-12-01269-s001.zip › images/HD_Node_Chart_VC.png]

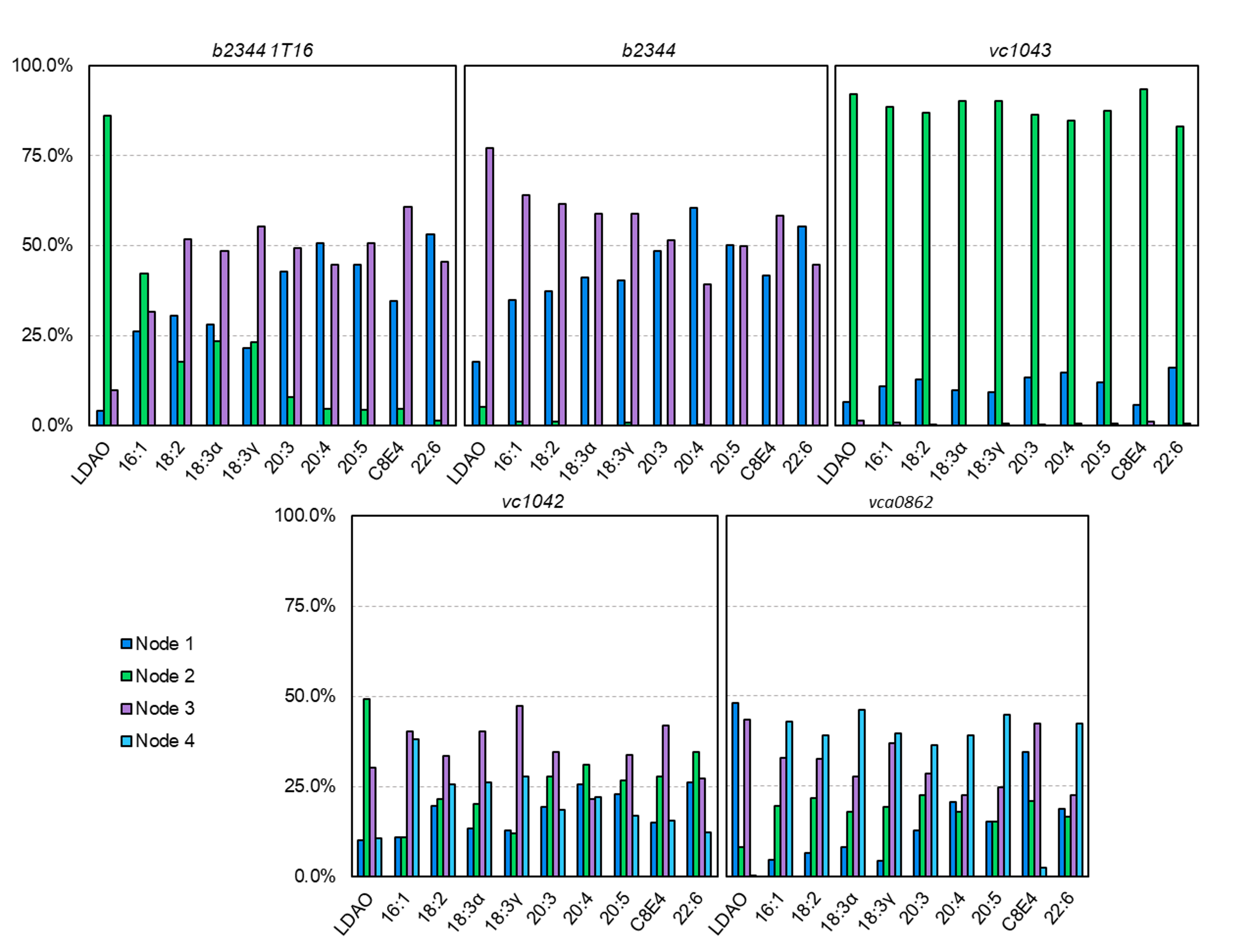

Supplement: Supplementary file 1 [file biomolecules-12-01269-s001.zip › images/HD_Node_Chart_vc.tif]

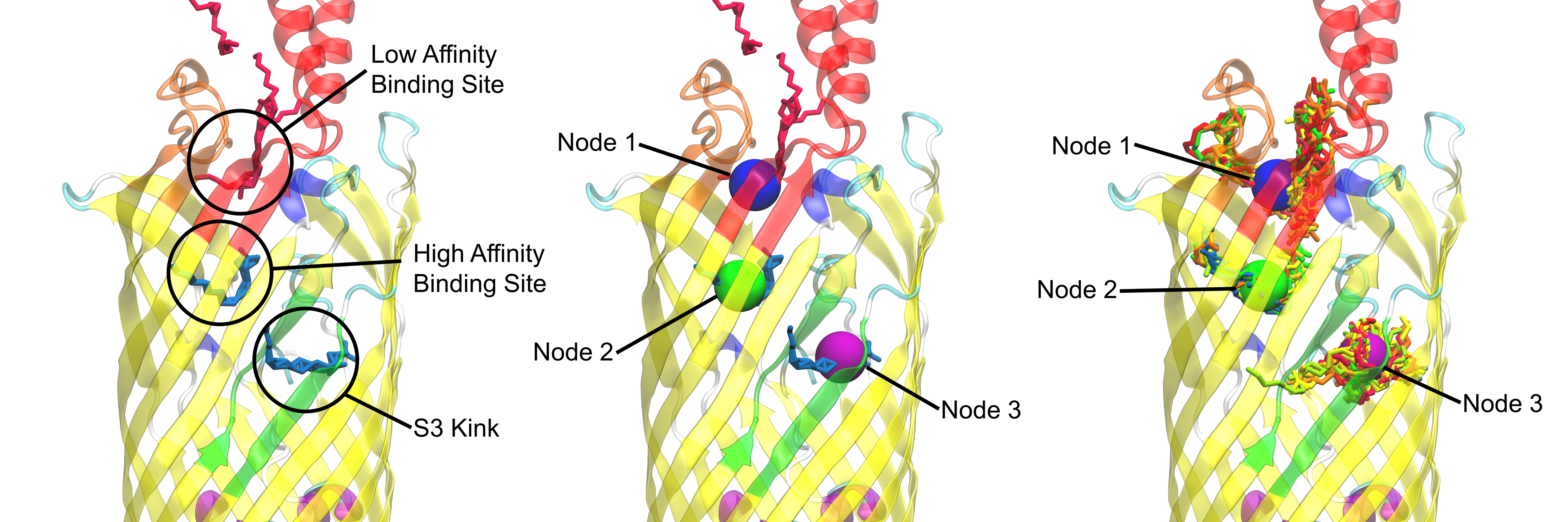

Supplement: Supplementary file 1 [file biomolecules-12-01269-s001.zip › images/HD_node_verification.png]

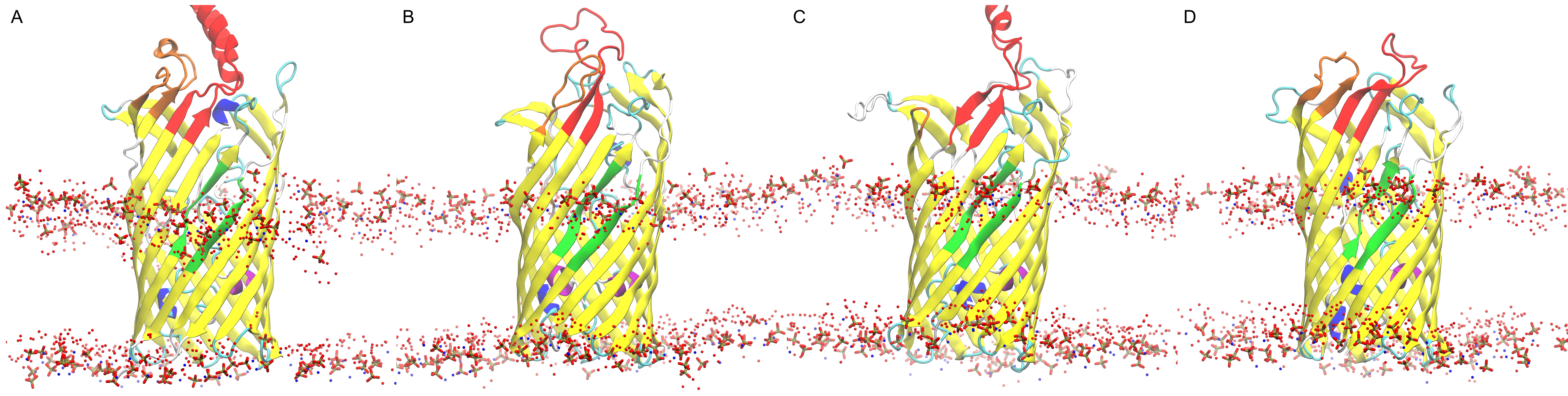

Supplement: Supplementary file 1 [file biomolecules-12-01269-s001.zip › images/HD_s3_layers.png]

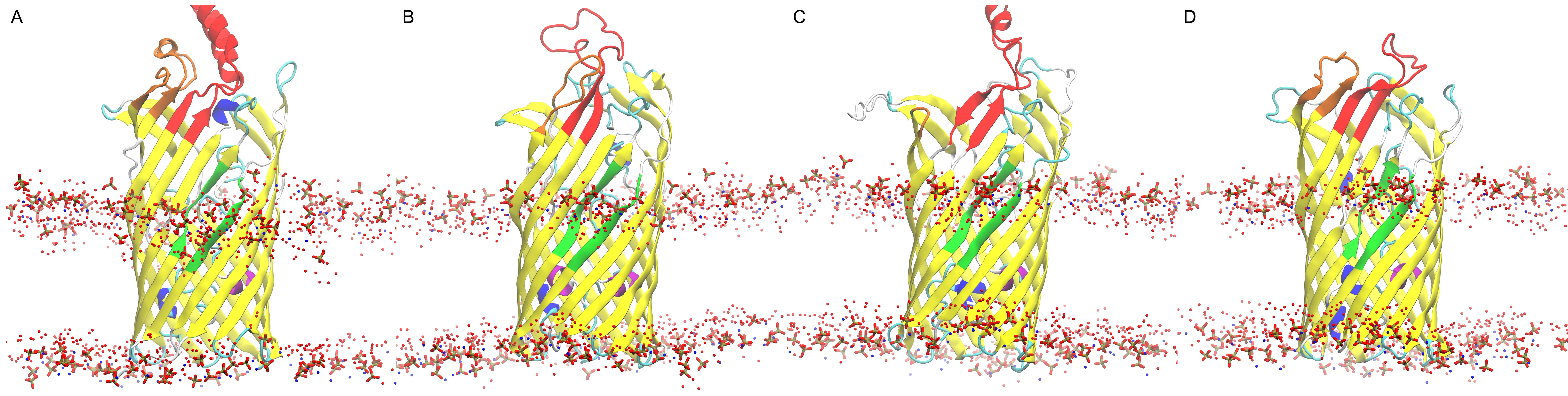

Supplement: Supplementary file 1 [file biomolecules-12-01269-s001.zip › images/HD_s3_layers.tif]

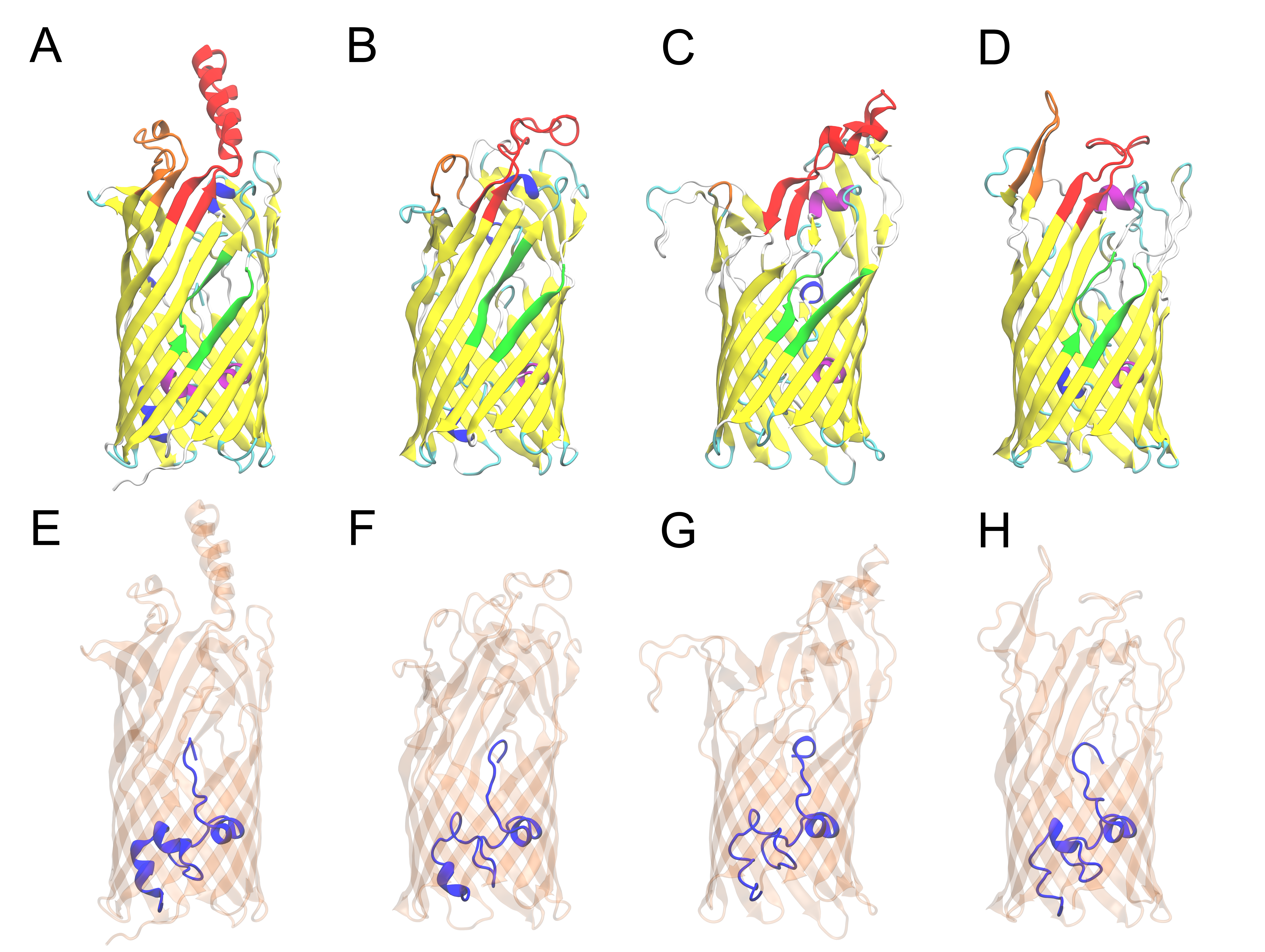

Supplement: Supplementary file 1 [file biomolecules-12-01269-s001.zip › images/HD_structures.png]

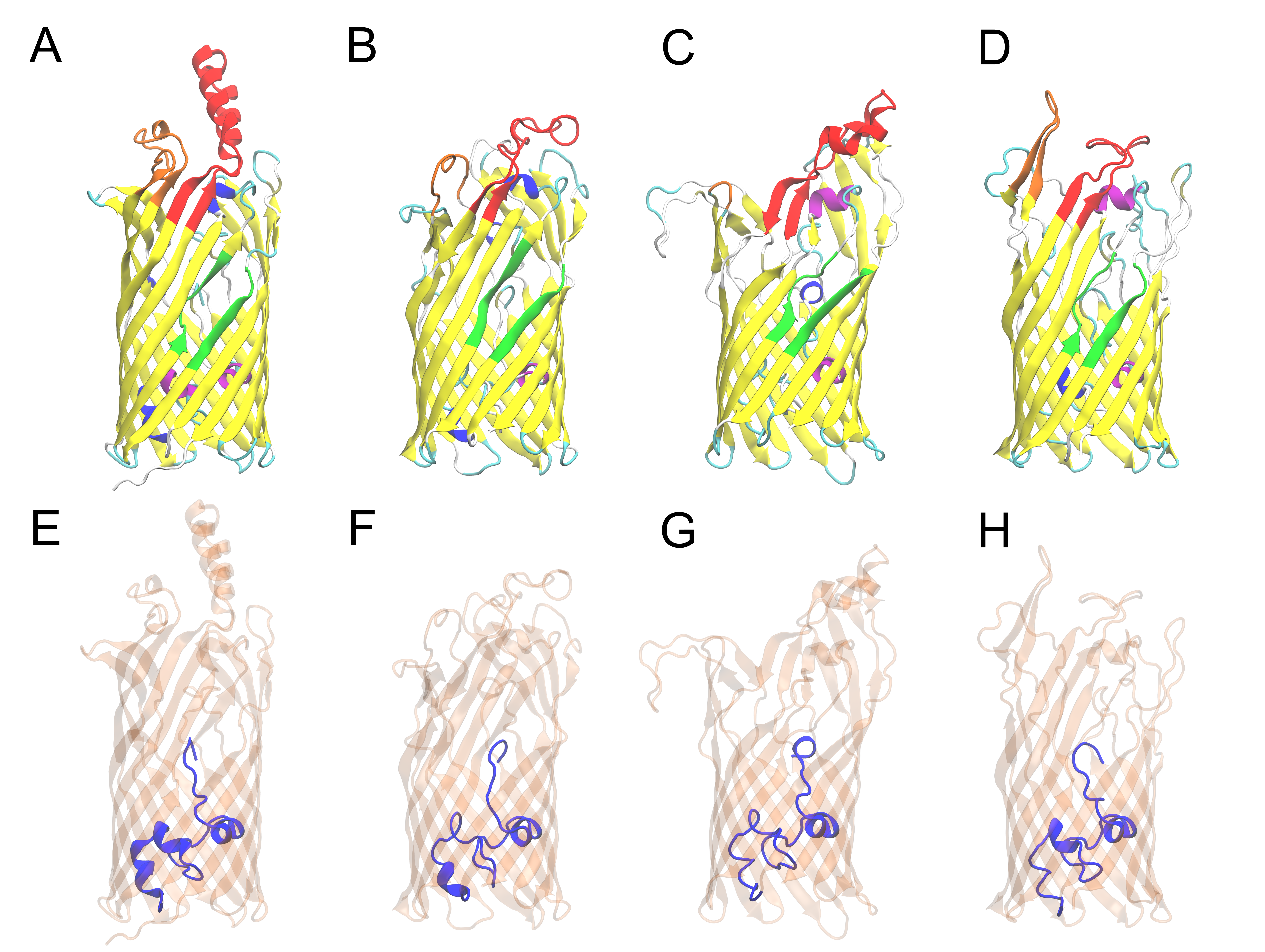

Supplement: Supplementary file 1 [file biomolecules-12-01269-s001.zip › images/HD_structures.tif]

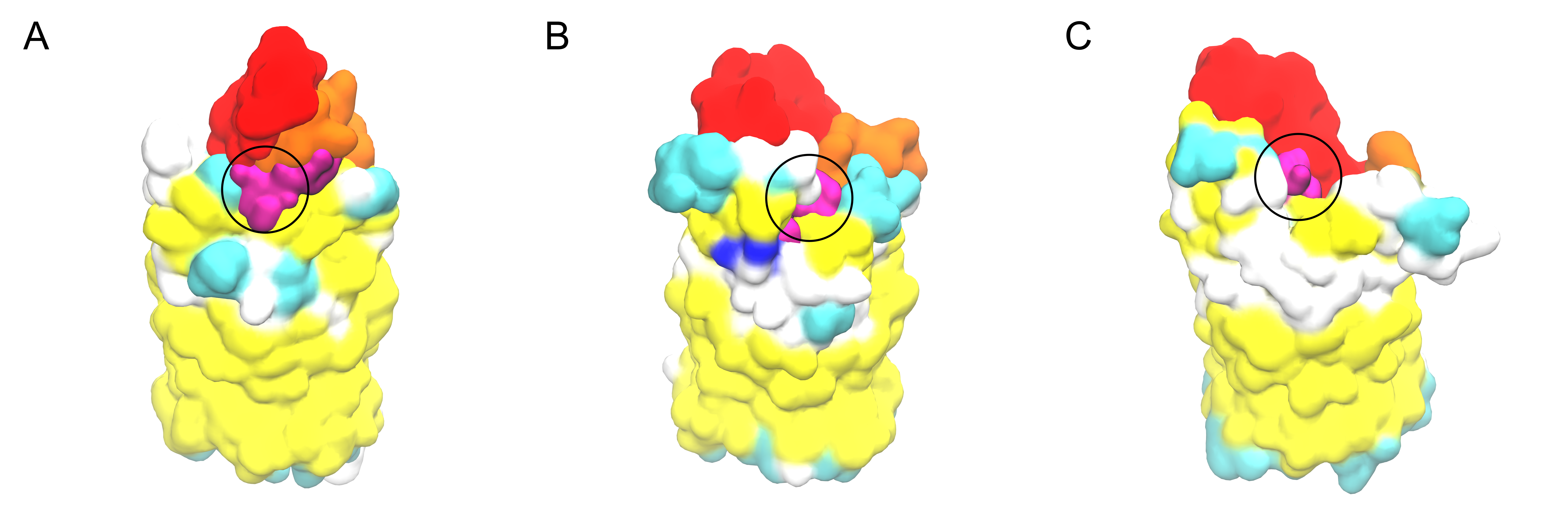

Supplement: Supplementary file 1 [file biomolecules-12-01269-s001.zip › images/HD_uptake_all.png]

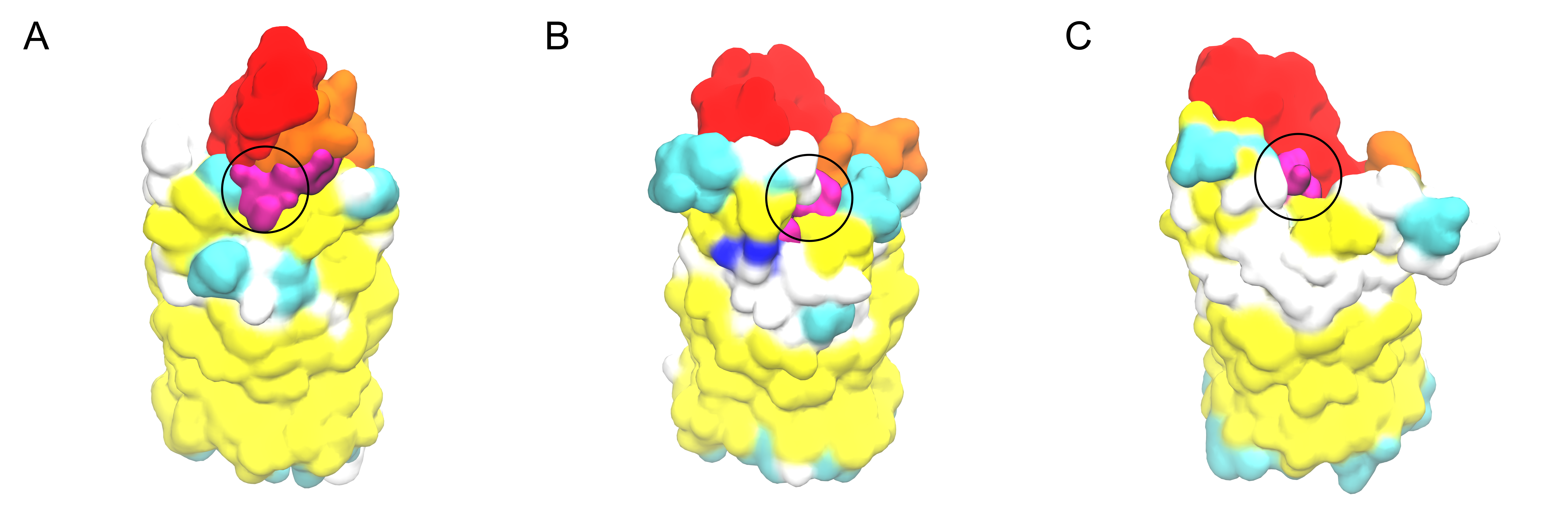

Supplement: Supplementary file 1 [file biomolecules-12-01269-s001.zip › images/HD_uptake_all.tif]

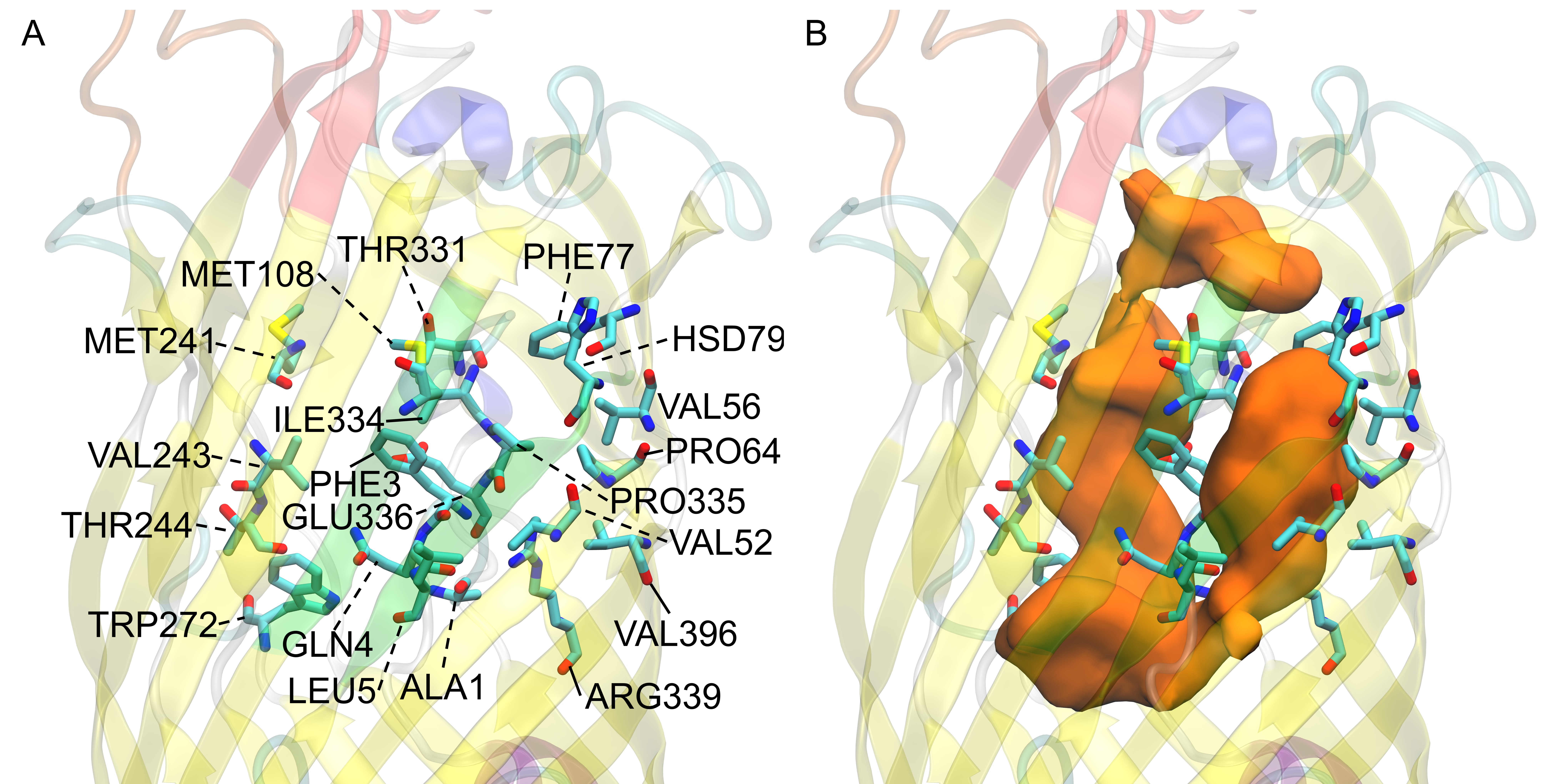

Supplement: Supplementary file 1 [file biomolecules-12-01269-s001.zip › images/HD_vc1042_channel.png]

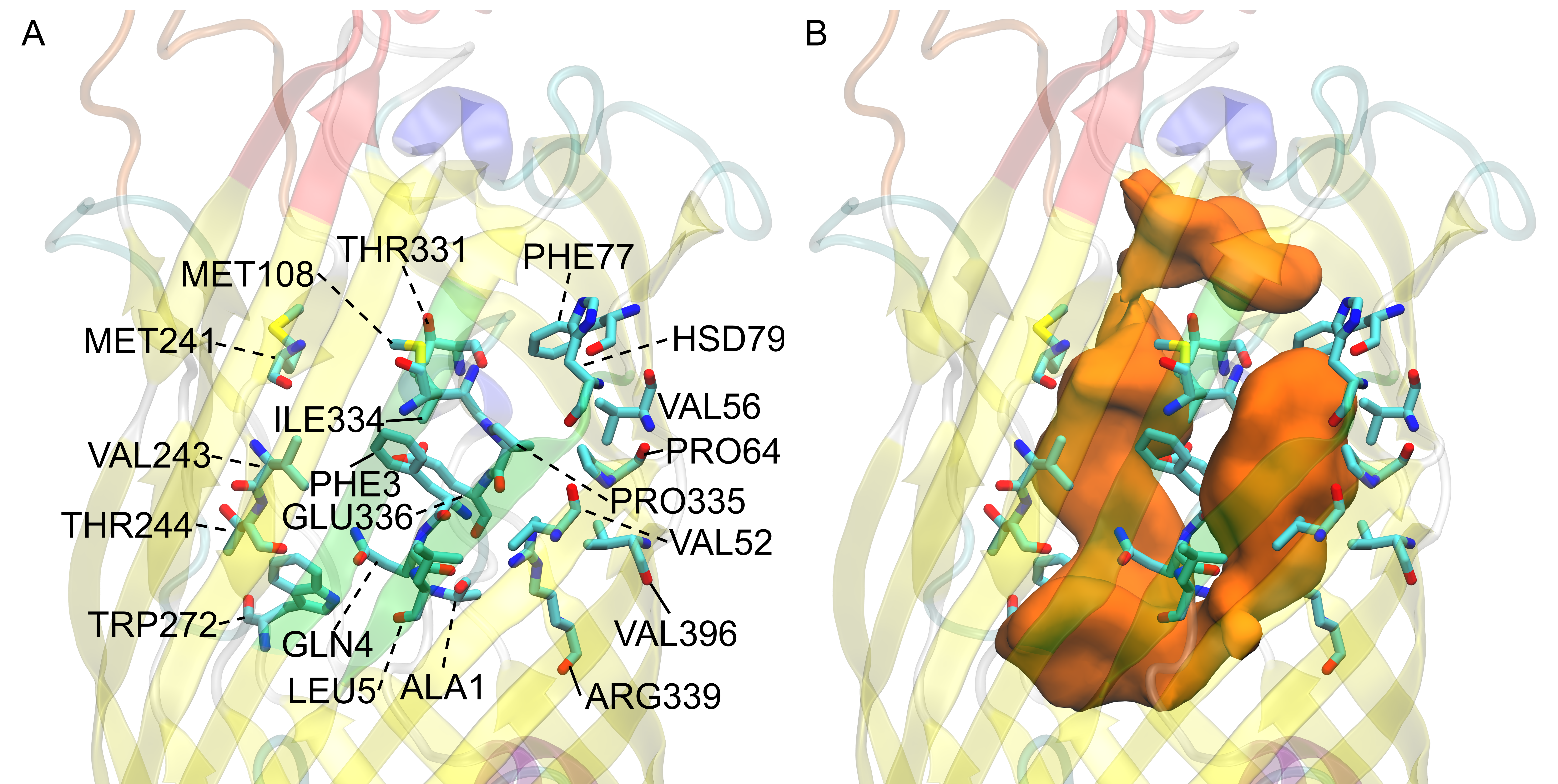

Supplement: Supplementary file 1 [file biomolecules-12-01269-s001.zip › images/HD_vc1042_channel.tif]

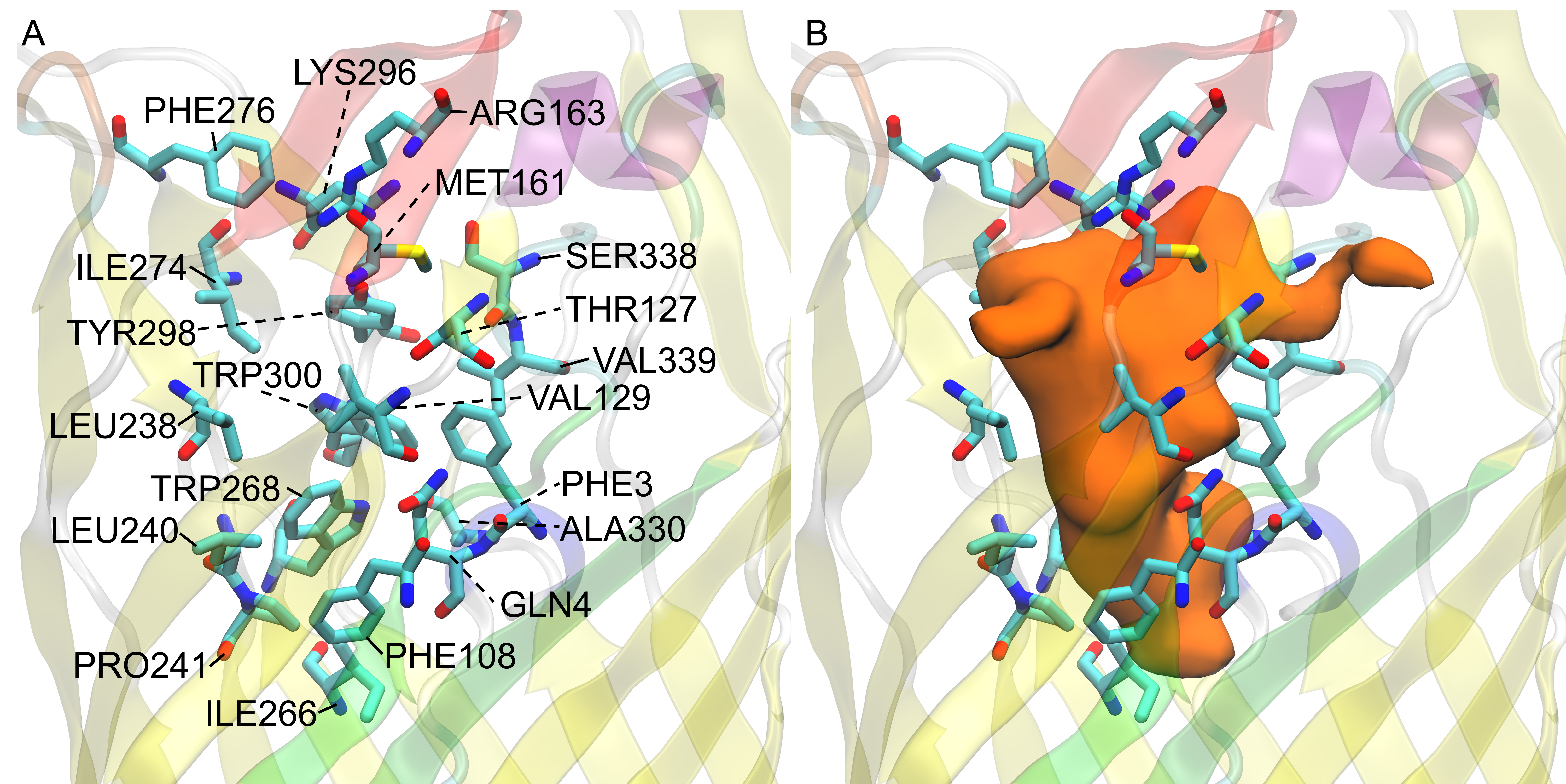

Supplement: Supplementary file 1 [file biomolecules-12-01269-s001.zip › images/HD_vc1043_channel.png]

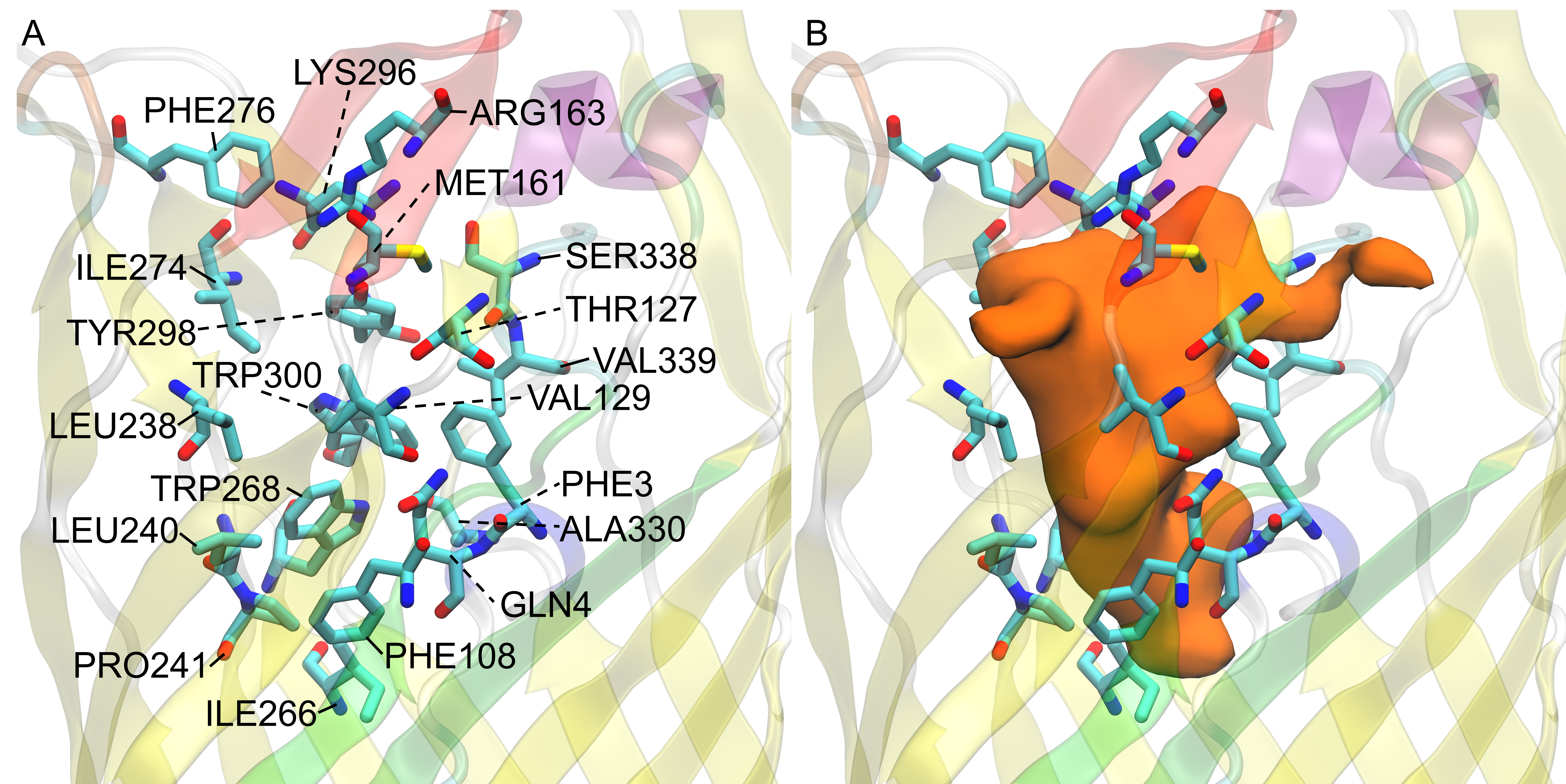

Supplement: Supplementary file 1 [file biomolecules-12-01269-s001.zip › images/HD_vc1043_channel.tif]

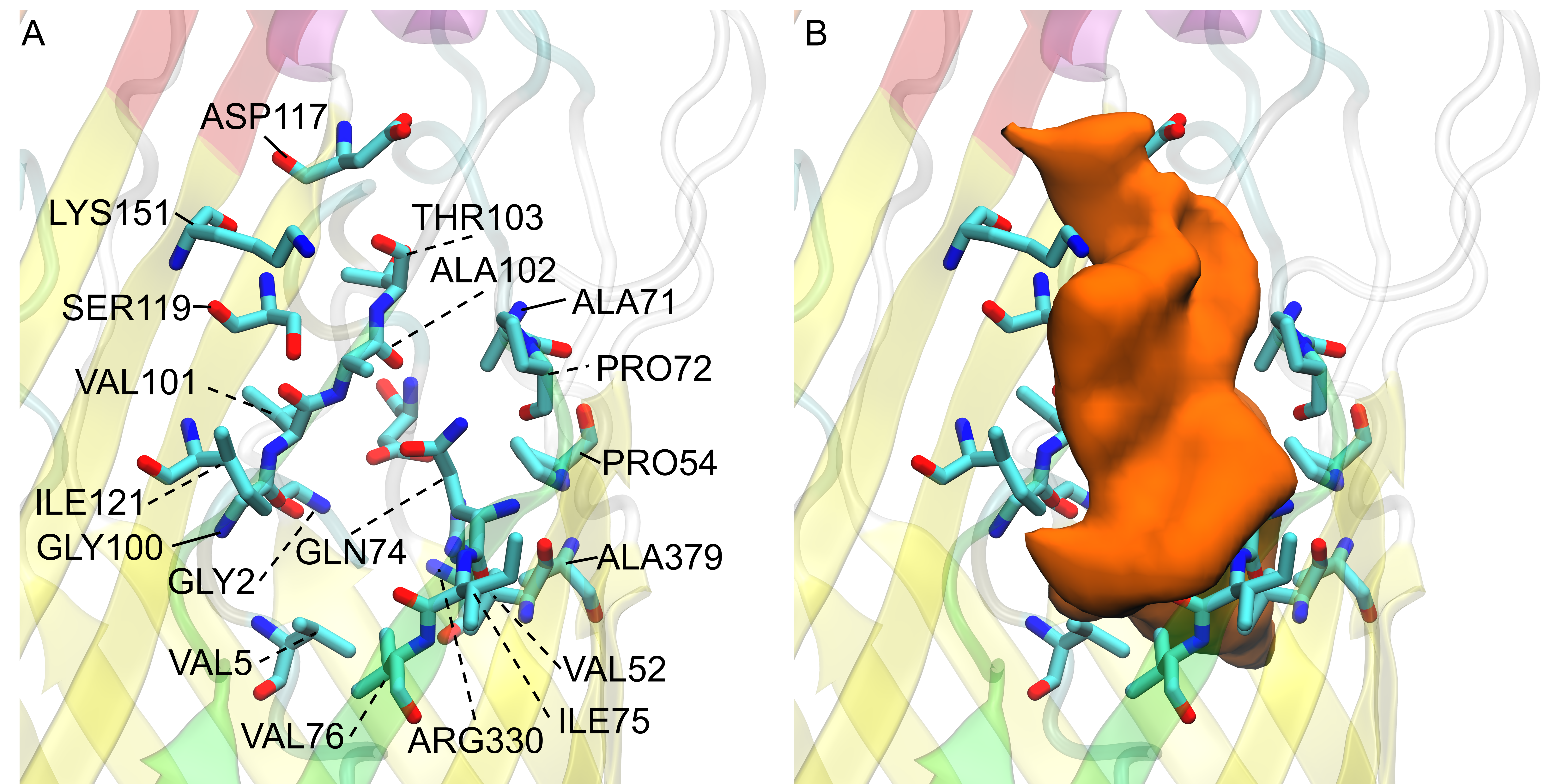

Supplement: Supplementary file 1 [file biomolecules-12-01269-s001.zip › images/HD_vca0862_channel.png]

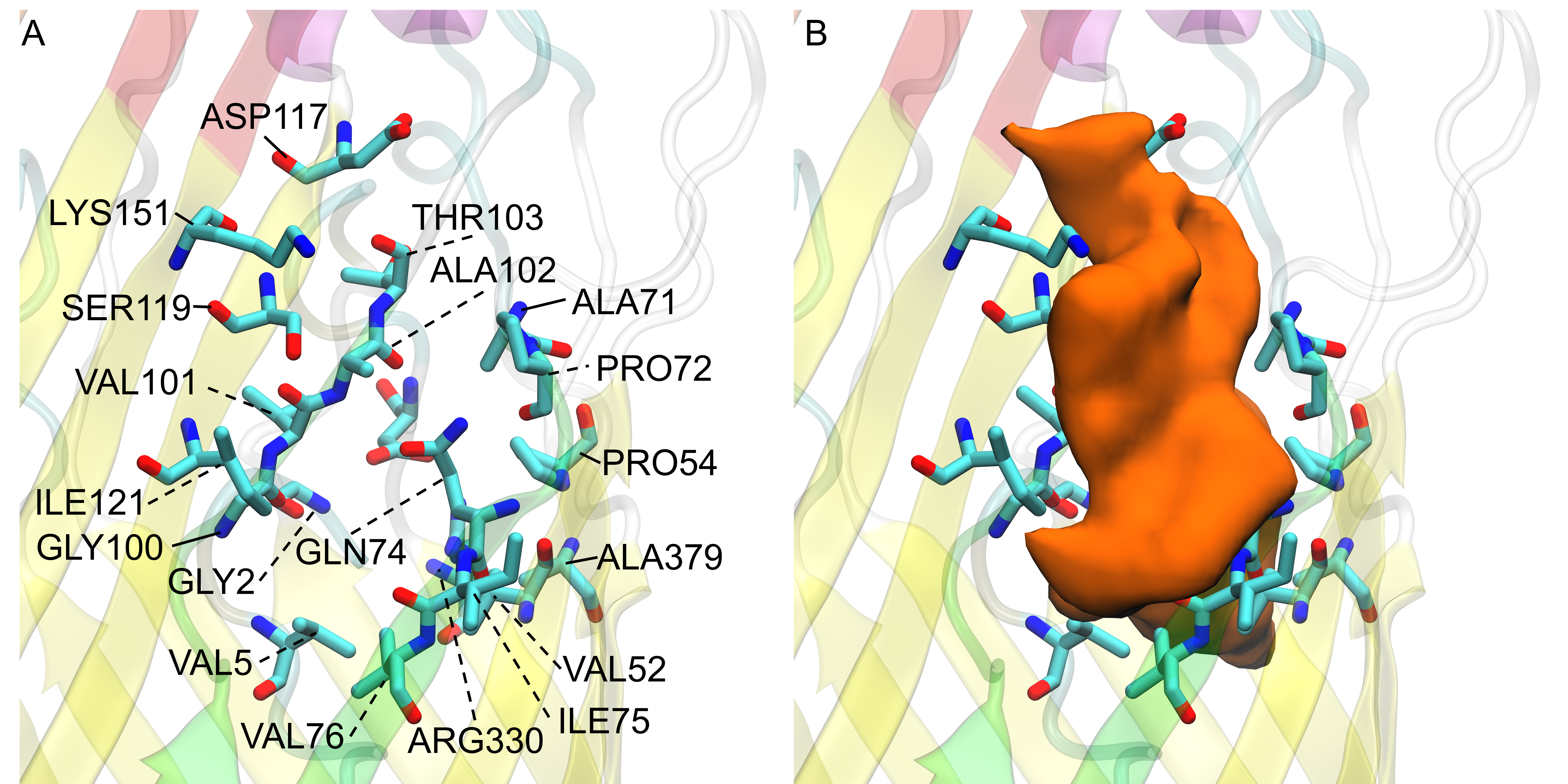

Supplement: Supplementary file 1 [file biomolecules-12-01269-s001.zip › images/HD_vca0862_channel.tif]

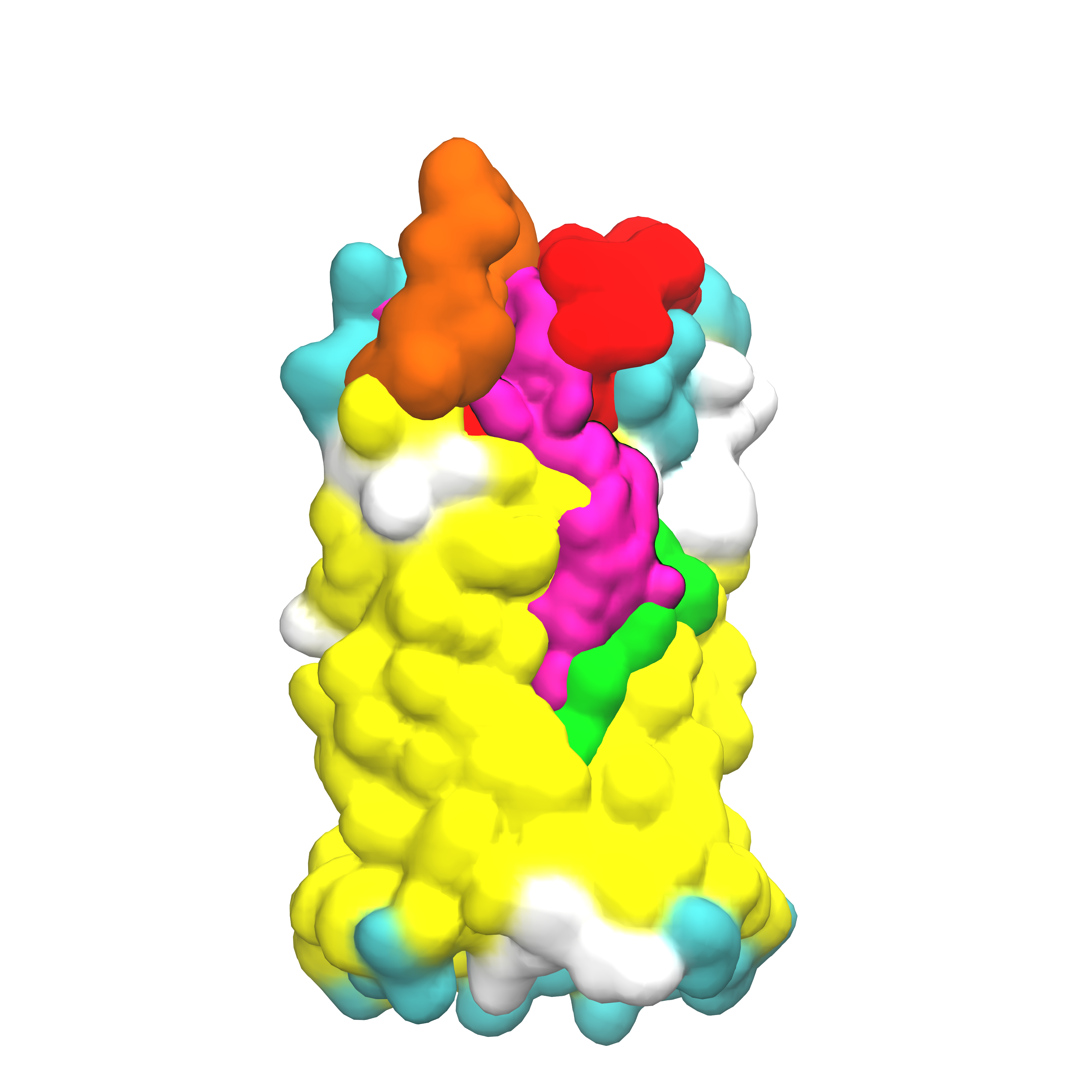

Supplement: Supplementary file 1 [file biomolecules-12-01269-s001.zip › images/HD_vca0862_surfaceS3.png]

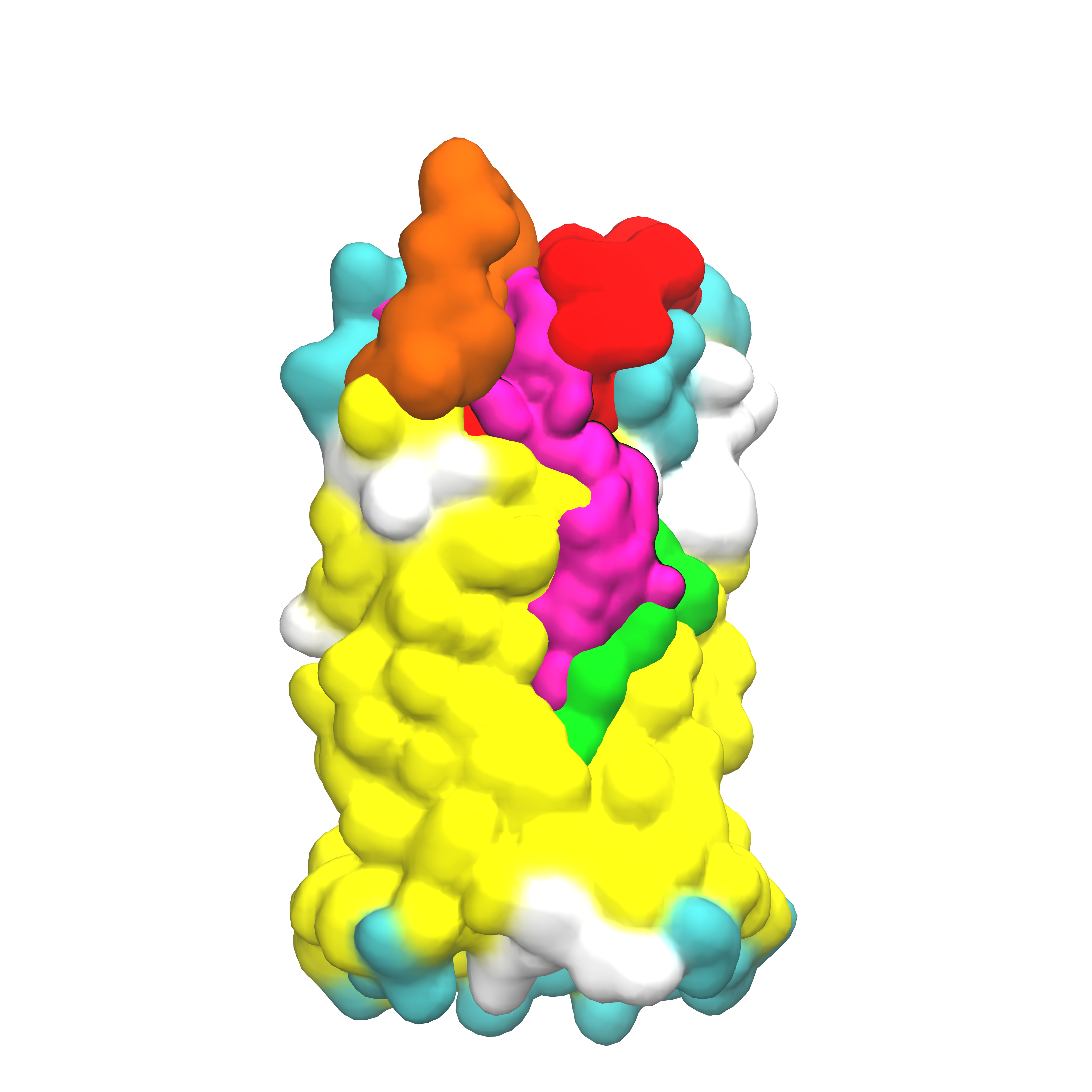

Supplement: Supplementary file 1 [file biomolecules-12-01269-s001.zip › images/HD_vca0862_surfaceS3.tif]

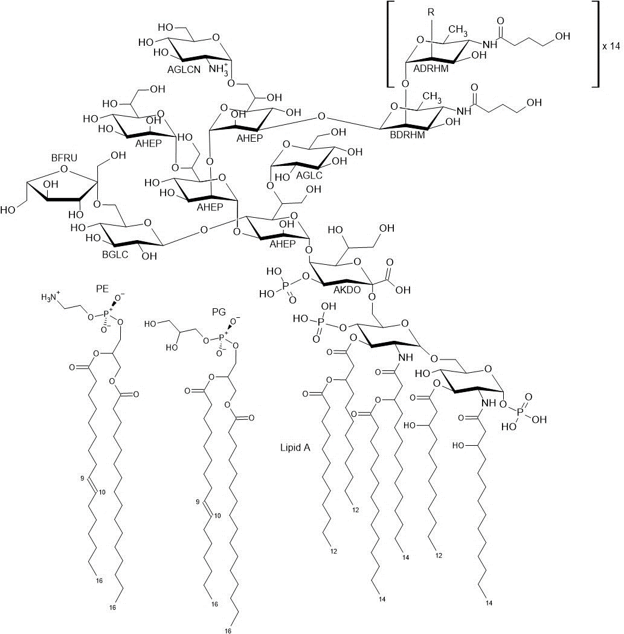

Supplement: Supplementary file 1 [file biomolecules-12-01269-s001.zip › images/LPS_Structures.png]

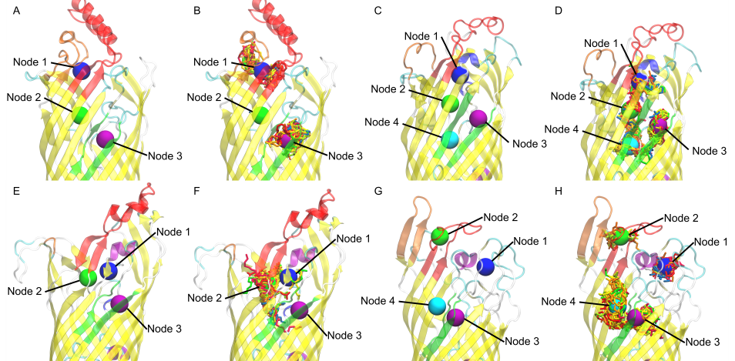

Supplement: Supplementary file 1 [file biomolecules-12-01269-s001.zip › images/nodes_vc.png]

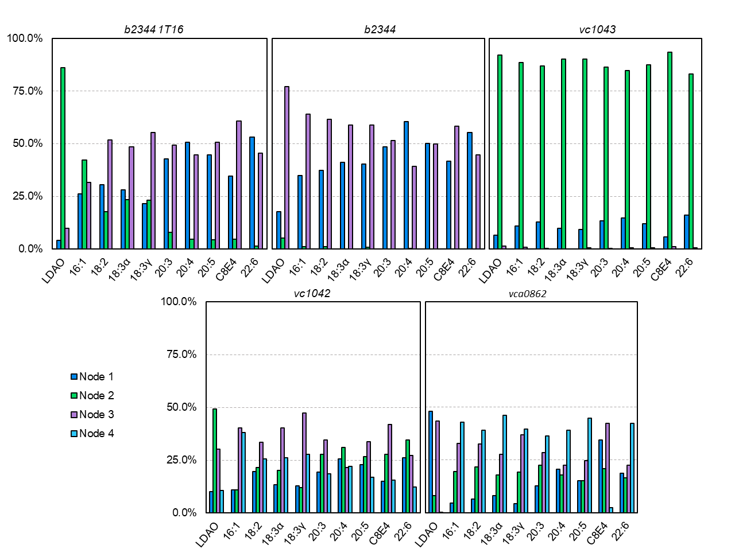

Supplement: Supplementary file 1 [file biomolecules-12-01269-s001.zip › images/Node_Chart_VC.png]

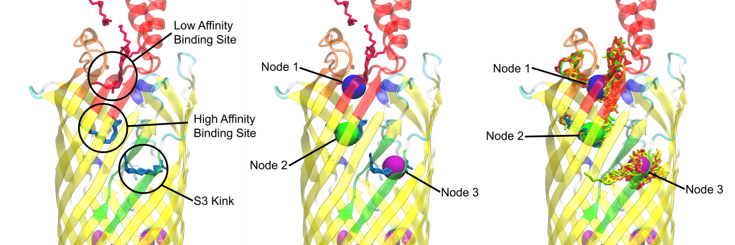

Supplement: Supplementary file 1 [file biomolecules-12-01269-s001.zip › images/node_verification.png]

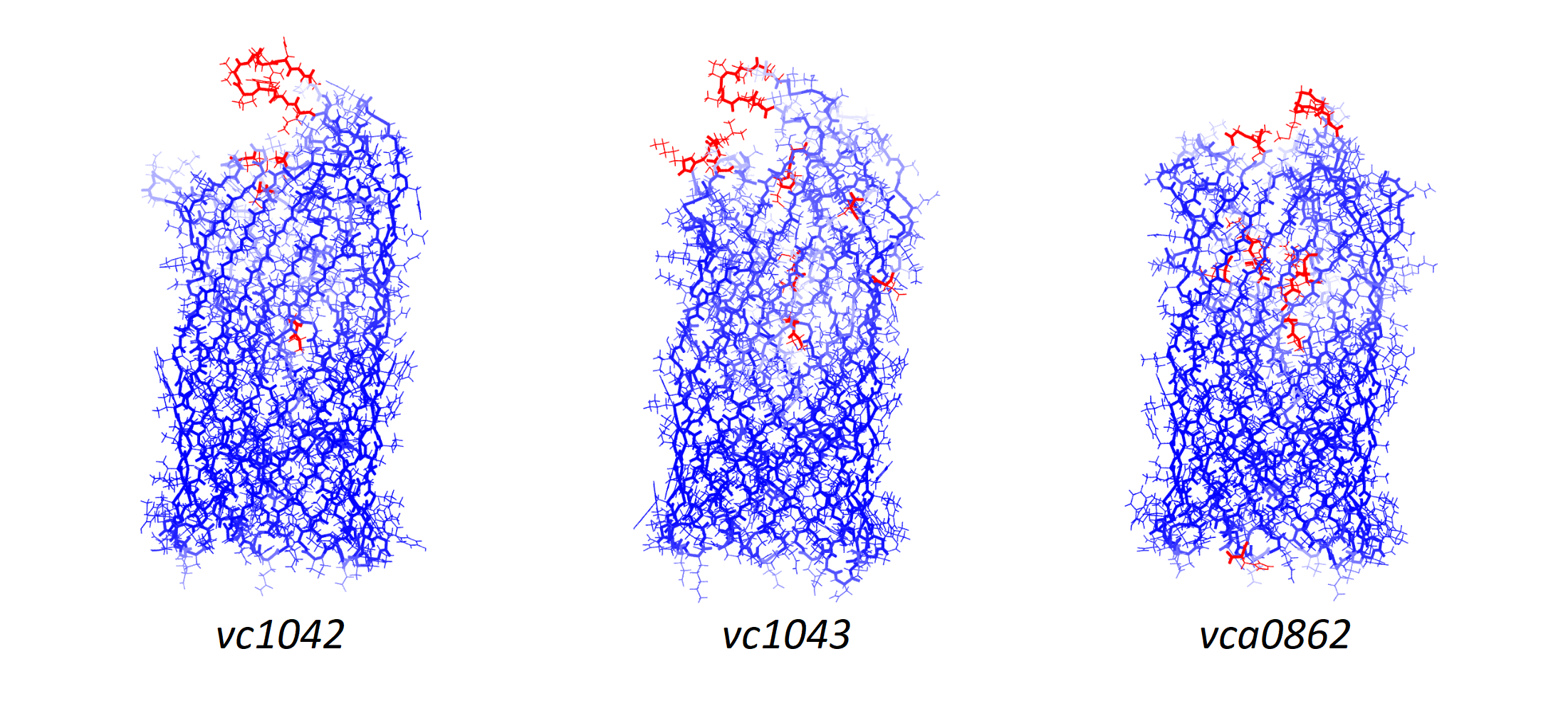

Supplement: Supplementary file 1 [file biomolecules-12-01269-s001.zip › images/PLDDT.png]

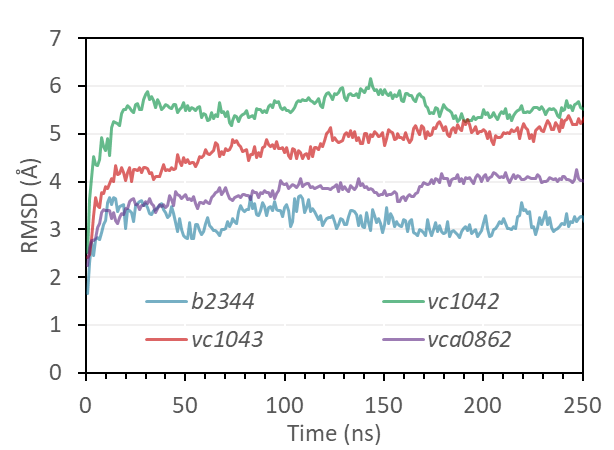

Supplement: Supplementary file 1 [file biomolecules-12-01269-s001.zip › images/RMSD.png]

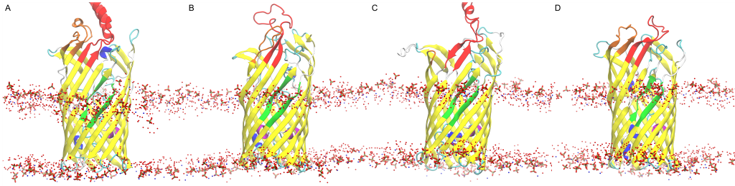

Supplement: Supplementary file 1 [file biomolecules-12-01269-s001.zip › images/s3_layers.png]

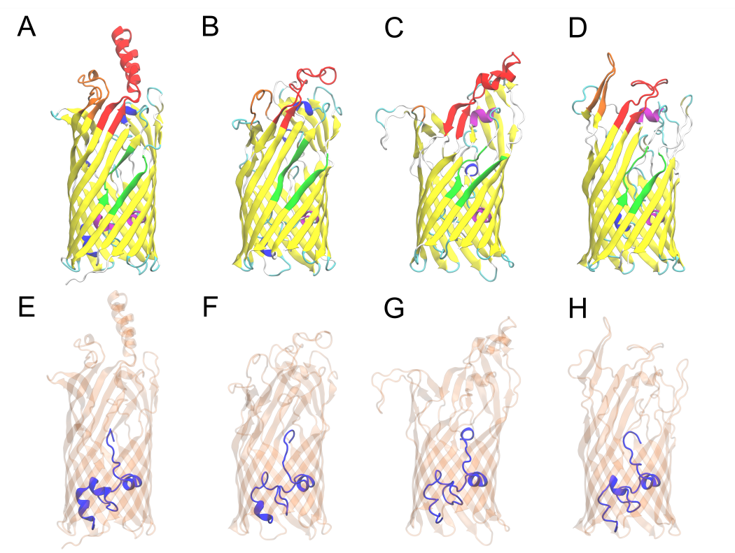

Supplement: Supplementary file 1 [file biomolecules-12-01269-s001.zip › images/structures.png]

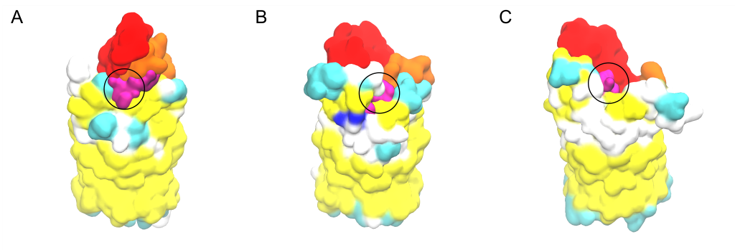

Supplement: Supplementary file 1 [file biomolecules-12-01269-s001.zip › images/uptake_all.png]

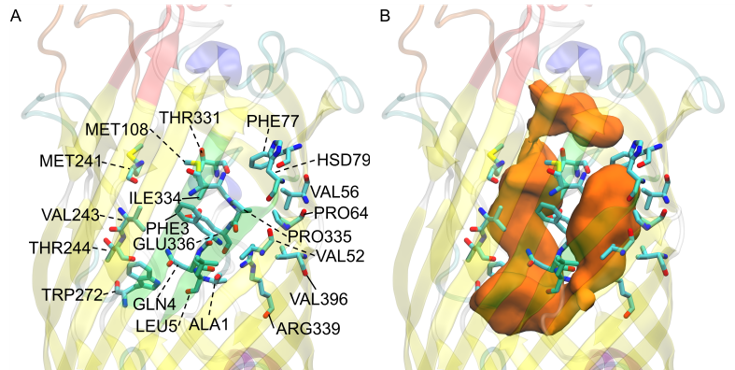

Supplement: Supplementary file 1 [file biomolecules-12-01269-s001.zip › images/vc1042_channel.png]

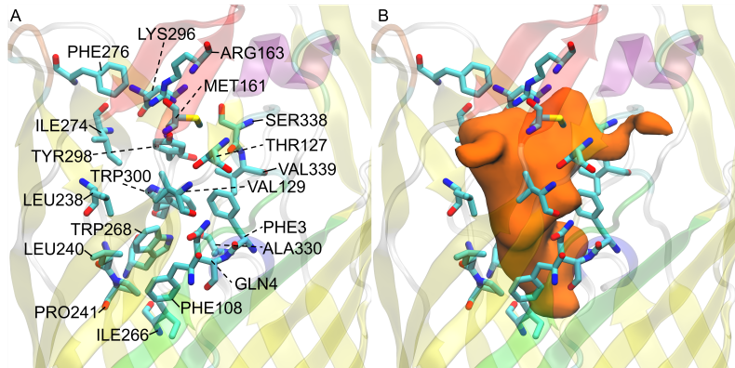

Supplement: Supplementary file 1 [file biomolecules-12-01269-s001.zip › images/vc1043_channel.png]

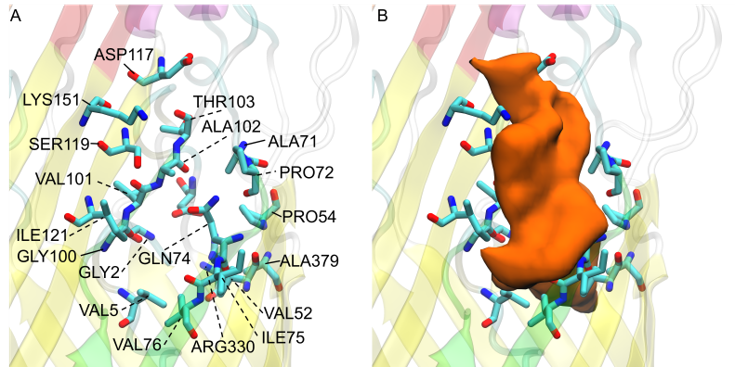

Supplement: Supplementary file 1 [file biomolecules-12-01269-s001.zip › images/vca0862_channel.png]

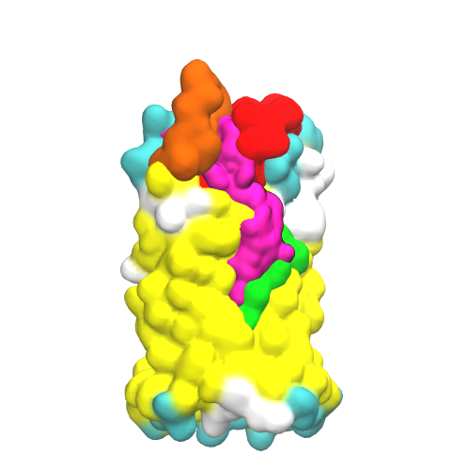

Supplement: Supplementary file 1 [file biomolecules-12-01269-s001.zip › images/vca0862_surfaceS3.png]
